# Juno and CD9 protein network organization in oolemma of mouse oocyte

**DOI:** 10.3389/fcell.2023.1110681

**Published:** 2023-08-10

**Authors:** Michaela Frolikova, Vishma Pratap Sur, Ivan Novotny, Michaela Blazikova, Jana Vondrakova, Ondrej Simonik, Lukas Ded, Eliska Valaskova, Lenka Koptasikova, Ales Benda, Pavla Postlerova, Ondrej Horvath, Katerina Komrskova

**Affiliations:** ^1^ Laboratory of Reproductive Biology, Institute of Biotechnology of the Czech Academy of Sciences, BIOCEV, Vestec, Czechia; ^2^ Light Microscopy Core Facility, Institute of Molecular Genetics of the Czech Academy of Sciences, Prague, Czechia; ^3^ Imaging Methods Core Facility at BIOCEV, Faculty of Science, Charles University, Vestec, Czechia; ^4^ Department of Veterinary Sciences, Faculty of Agrobiology, Food and Natural Resources, University of Life Sciences Prague, Prague, Czechia; ^5^ Department of Zoology, Faculty of Science, Charles University, Prague, Czechia

**Keywords:** oocyte, Juno, CD9, oolemma compartments, protein interaction, STED, docking, MD simulation

## Abstract

Juno and CD9 protein, expressed in oolemma, are known to be essential for sperm-oocyte binding and fusion. Although evidence exists that these two proteins cooperate, their interaction has not yet been demonstrated. Here in, we present Juno and CD9 mutual localization over the surface of mouse metaphase II oocytes captured using the 3D STED super-resolution technique. The precise localization of examined proteins was identified in different compartments of oolemma such as the microvillar membrane, planar membrane between individual *microvilli*, and the membrane of *microvilli*-free region. Observed variance in localization of Juno and CD9 was confirmed by analysis of transmission and scanning electron microscopy images, which showed a significant difference in the presence of proteins between selected membrane compartments. Colocalization analysis of super-resolution images based on Pearson’s correlation coefficient supported evidence of Juno and CD9 mutual position in the oolemma, which was identified by proximity ligation assay. Importantly, the interaction between Juno and CD9 was detected by co-immunoprecipitation and mass spectrometry in HEK293T/17 transfected cell line. For better understanding of experimental data, mouse Juno and CD9 3D structure were prepared by comparative homology modelling and several protein-protein flexible sidechain dockings were performed using the ClusPro server. The dynamic state of the proteins was studied in real-time at atomic level by molecular dynamics (MD) simulation. Docking and MD simulation predicted Juno-CD9 interactions and stability also suggesting an interactive mechanism. Using the multiscale approach, we detected close proximity of Juno and CD9 within microvillar oolemma however, not in the planar membrane or *microvilli*-free region. Our findings show yet unidentified Juno and CD9 interaction within the mouse oolemma protein network prior to sperm attachment. These results suggest that a Juno and CD9 interactive network could assist in primary Juno binding to sperm Izumo1 as a prerequisite to subsequent gamete membrane fusion.

## 1 Introduction

In mammals, fertilization is characterized by a cascade of protein-protein interactions between oocyte and sperm, however, the precise molecular mechanism of gamete binding and fusion has not yet been demonstrated. In oocyte, the glycophosphatidylinositol (GPI) anchored protein Juno (Bianchi et al., 2014) and tetraspanin family member CD9 (Kaji et al., 2000; Le Naour et al., 2000; Miyado et al., 2000) were identified to be essential for mammalian fertilization. In human, Juno was also reported to interact with sperm Izumo1 (Bianchi et al., 2014), and recently was proposed to be superseded during human gamete fusion by a newly identified human oocyte receptor FcRL3, named MAIA (Vondrakova et al., 2022). Nevertheless, the role of CD9 in the membrane protein network remains unclear. Despite the close proximity between Juno and CD9 proteins in oolemma, their interaction has not been shown. The initial contact between a sperm head plasma membrane and oolemma is probably facilitated by tetraspanin proteins (Bianchi and Wright, 2020) and mediated via mutual interaction between Juno (Bianchi et al., 2014) and its sperm receptor Izumo1 (Inoue et al., 2005). Tetraspanin proteins contribute to membrane compartment organization and via *cis* interaction the multimolecular web and protein clusters are formed (Jankovičová et al., 2020) that include integrins (Ziyyat et al., 2006) or EWI proteins (Sala-Valdés et al., 2006; Runge et al., 2007; Umeda et al., 2020). Importantly, CD9 deficiency resulted in severe fertility malfunction in female mice (Kaji et al., 2000; Le Naour et al., 2000; Miyado et al., 2000) reviewed in (Bianchi and Wright, 2016) and evidence was provided, that Juno localization is abnormal in CD9^−/−^ oocytes (Inoue et al., 2020). Furthermore, distribution of CD9 is not uniform within oolemma and it is expressed only on *microvilli*, where CD9 is critical for influencing their number and shape (Runge et al., 2007; Benammar et al., 2017). The CD9 crystal structure revealed a molecule of highly asymmetric cone-like shape which is believed to be responsible for the generation of membrane curvature in the crystalline lipid layers (Umeda et al., 2020). Additionally, this could explain CD9 localization in membrane compartments with high curvature such as *microvilli*. The large extracellular loop of CD9 is most likely responsible for the critical role of CD9 in sperm-oocyte fusion (Zhu et al., 2002; Umeda et al., 2020). In mouse, the oocyte surface is not homogenous, and it is distinguished to a *microvilli*-free region covering the meiotic spindle and *microvilli*-rich region which facilitates sperm binding and fusion (Shalgi and Phillips, 1980; Yanagimachi, 1994). In addition to organizing *microvilli* formation, CD9 may participate in generating fusion competent sites (Jégou et al., 2011), which is supported by the accumulation of CD9 in the intercellular contact area after mutual binding of Izumo1 and Juno (Chalbi et al., 2014). This suggests the interaction of Juno and CD9 (Chalbi et al., 2014) and possible CD9 involvement in the organization of Juno localization within oolemma regions (Bianchi and Wright, 2016; Inoue et al., 2020). The role CD9 plays is supported by its participation in compartmentalization of oolemma and by the evidence that Juno is not well expressed in *microvilli*-free regions (Inoue et al., 2020). Importantly, CD9 is also included in molecular cargo carried by extracellular vesicles of unfertilized mouse oocytes (Miyado et al., 2008). Although, partial co-localization of Juno and CD9 was observed using Airy scan super-resolution imaging, precise Juno localization in the membrane compartments of *microvilli* rich regions is not known (Mori et al., 2021).

Protein structure and protein-protein interactive interfaces provide valuable information about the function of protein networks and presents a novel approach for microscopical data. It should be noted that, to obtain protein 3D structural prediction from amino acid sequences using experimental techniques like X-ray crystallography or NMR is challenging, time consuming, expensive, and not successful with certain proteins especially transmembrane proteins (Vyas et al., 2012). In the absence of experimental protein crystal structure, computationally derived structures can be generated using comparative modeling methods, or free modeling techniques. Currently, there is no crystal structure of mouse CD9 molecule available, therefore the computer program based comparative protein structural modelling software MODELLER was used for CD9 structure prediction (Webb and Sali, 2016). Protein-protein interactions trigger the molecular mechanism of most biological processes, their prediction is important for understanding various biological events and can be carried out using ClusPro (Kozakov et al., 2017); ZDOCK (Pierce et al., 2014); HADDOCK (de Vries et al., 2010) or HexServer (Macindoe et al., 2010). The experimental Juno-CD9 results were subsequently followed by protein 3D structural modelling to study individual protein structural and functional roles in biological systems, and their mutual interactions. To better understand the protein and protein-protein orientation within the membrane, a protein-membrane model structural prediction was performed followed by protein-protein side chains docking, to recognize the Juno-CD9 interacting pose and interacting residues in their dynamic state. To reveal Juno and CD9 motion, the protein-protein MD simulation was performed to identify the interaction stability, structural deviations, and real-time secondary structure alterations in the Juno-CD9 complex.

In this study, we aimed to clarify questions regarding the proximity of Juno and CD9 and their possible interaction within the same protein network in mouse oolemma. Using 3D STED super-resolution microscopy, electron microscopy and follow-up image analysis we detected and verified Juno and CD9 colocalization in the membrane of *microvilli*. Furthermore, proximity ligation assay (PLA) identified Juno and CD9 close proximity association and co-immunoprecipitation (co-IP) followed by mass spectrometry (MS) confirmed their interaction in HEK293T/17 transfected cell line. Molecular docking, protein-membrane structural prediction, and MD simulation was used to tract protein-protein interaction, their structural position in the biological membrane, and protein conformational changes at atomic level over time. Molecular docking and protein-membrane modeling indicated the most likely biologically relevant interaction mode and MD simulation confirmed Juno-CD9 mutual interactions and the stability of their complex. It also indicated an interactive mechanism and function with respect to Juno and CD9 motion.

## 2 Materials and methods

### 2.1 Animal source and ethics approval

Inbred C57BL/6J female mice were housed in a breeding colony of the Laboratory of Reproduction, IMG animal facilities, Institute of Molecular Genetics of Czech Academy of Science. Food and water were supplied *ad libitum*. The female mice used for all experiments were healthy 23–26 days old with no signs of stress or discomfort. All the animal procedures and experimental protocols were approved by the Animal Welfare Committee of the Czech Academy of Sciences (Animal Ethics Number 66866/2015-MZE-17214, 18 December 2015).

### 2.2 Oocytes collection

Female mice were hormonally stimulated with 5UI PMSG, pregnant mare serum gonadotropine (Folligon^®^, Intervet International B.V., Netherlands), at 15:00 (eighth hour of light cycle) on the first day of protocol. 5UI of hCG, human Chorionic Gonadotropin (CG10, Sigma-Aldrich^®^, MI, USA), were applied to mice at 13:00 third day of protocol (46th hour after using PMSG). After 12 h, females started ovulating. At 09:00 on the fourth day of protocol, female mice were sacrificed by cervical dislocation and both ampullas of fallopian tube were isolated and placed in preheated M2 medium (M7167, Sigma-Aldrich^®^). Cumulus-oocytes complex (COC) was released into M2 medium by ampulla tearing. In the next step, for releasing cumulus cells, COC was transferred into fresh 100 mL drop of M2 medium with hyaluronidase (concentration 0.1 mg/mL) (Hyase, from bovine testes, H4272, Sigma-Aldrich^®^), covered with high viscous paraffin oil (P14501, Carl Roth, Germany) and left in the incubator (set on 37°C, 5% CO_2_) for 10 min. Followed by washing, the cumulus-free oocytes were transfer into drop of Tyrode’s solution (T1788, Sigma-Aldrich^®^) to remove *zona pellucida*.

### 2.3 STED microscopy

#### 2.3.1 Oocyte preparation

The zona-free oocytes were fixed with 3.7% paraformaldehyde (P6184, Sigma-Aldrich^®^) for 20 min and washed 2 × in 1% bovine serum albumin (BSA) (A-1933-25G, Sigma-Aldrich^®^) in PBS (phosphate-buffered saline). The oocytes were incubated overnight in 4 °C in drop of primary antibodies rat anti-CD9 (KMC8.8) (sc18869, Santa Cruz Biotechnology, Inc., TX, USA) diluted 1:50 in 1% BSA and rabbit polyclonal anti Folate receptor 4 (Juno) (abx102438, Abbexa^®^, UK) diluted 1:50 in 1% BSA followed by secondary antibody anti-rat IgG Abberior STAR 635P (Abberior GmbH, Germany) and anti-rabbit IgG Abberior STAR 580 diluted 1:100 in 1% BSA were incubated for 1 h in room temperature (RT) and wash 2 × in 1% BSA in PBS. The finalization of the oocyte sample preparation was proceeded by a gentle alcohol-based dehydration of the specimen followed with gradual transfer into 2,2′-Thiodiethanol (TDE) based mounting medium AD-MOUNT C (ADM-009, ADVI, Czech Republic). To preserve the fragile oocyte structure, for the mounting between cover-glass and slide we applied 150 µm thick mounting spacers AD-SEAL (ADS-18-10150-20, ADVI).

#### 2.3.2 Oocyte capturing

The STED super-resolution acquisition of oocytes was performed on a Leica TSC SP8 STED 3X microscope equipped with a pulse white light laser (WLL2) (Light Microscopy Core Facility, IMG CAS, Prague, Czech Republic) for the excitation and a pulse 775 nm laser for emission depletion. Images were acquired as *Z*-stacks in 3D STED imaging mode with settings: HC PL APO 100×/1.4 OIL STED WHITE CS2, oil n = 1.518, pinhole 0.6 AU according to the excitation wavelength 580 nm or 640 nm, pulsed 775 nm depletion laser with 60% 3D STED, detection was on HyD in photon counting mode, emission interval 587–617 nm or 647–728 nm respectively. Super-resolution images were finalized by deconvolution using the STED option module in Huygens Professional software (Scientific Volume Imaging, NL; version 20.10). For the analysis, top and bottom segments for each oocyte were acquired (see 2.5 Colocalization analysis). For the visualization, the entire oocyte was captured.

#### 2.3.3 Colocalization analysis of STED images

The super-resolved images of mouse oolemma top and bottom segments were segmented according to CD9 labelling. 2D sections (XY) were processed separately. We used edge detection and morphological operations to define the oocyte border and to select the first 60 layers (in pixels) starting from the envelope reaching inside toward the geometrical center of each section. The layers represented a border of 2.4 µm in thickness. Pearson’s correlation coefficient between CD9 and Juno labelling was calculated for each section in the selected layers and its average value along the *Z*-direction and standard deviation was computed. The average value of Pearson’s correlation coefficient was measured for 8 oocytes (a top and bottom segment for seven oocytes and only bottom for one oocyte were analyzed).

Manders’ correlation coefficients were evaluated using the same segmented areas for each oolemma top and bottom segment. The threshold was set up according to the automatic threshold algorithm (default, a variation of the IsoData algorithm) in Fiji (Schindelin et al., 2012). Coefficient M1 corresponds to the green channel (Juno) over red channel (CD9) overlap, while M2 to the red channel over green channel overlap. The values represent an average value along the *Z*-direction. The difference between the overall average value of M1 *versus* M2 was evaluated by *t*-test. Before testing, the normality of data was confirmed by Anderson–Darling test. The complete procedure was programmed in MATLAB (version 2019b, The MathWorks^®^, Natick, MA, USA).

Further quantification of Manders’ correlation coefficients in separated regions of planar membrane and microvillar regions was evaluated using Fiji software (Schindelin et al., 2012). A macro was used for automated segmentation of 40 cropped 2D regions of the oocyte, with a minimum border length of 10 µm. The macro is based on the default thresholding, skeletonization, and morphological operations to select regions corresponding to planar membrane (PlnM) or *microvilli* (MvM), with exclusion of the questionable areas below the *microvilli*. Manders’ coefficients M1 (green channel (Juno) over red channel (CD9) overlap and M2 (red channel over green channel overlap) were calculated separately for PlnM and MvM, using the same threshold for all cropped oocyte regions. The Mann–Whitney test (GraphPad Prism 9.3.1) was used to analyze the differences between the coefficients, with a *p*-value equal or lower than 0.05 considered significant (*p* ≤ 0.05*; 0.01**; 0.001***).

### 2.4 Electron microscopy

#### 2.4.1 Transmission electron microscopy (TEM)

The oocytes were prepared in the same way as described above until the primary antibodies were applied. The oocytes were then labelled for 1 h at RT with 6 nm or 12 nm gold-conjugated IgG secondary antibodies (Jackson ImmunoResearch, UK): 6 nm anti-rat (112-195-167, Jackson ImmunoResearch) diluted 1:30 in 1% BSA and 12 nm anti-rabbit (111-205-144, Jackson ImmunoResearch) diluted 1:30 in 1% BSA. After a brief wash, the oocytes were fixed with 3% glutaraldehyde (16220, Electron Microscopy Sciences, PA, USA) in 1% BSA in PBS pH 7.4 for 1 h at RT and for next at least 72 h at 4°C. The samples were cold-PBS washed 3 × 15 min at RT, post-fixed with 1% aqueous osmium tetroxide (#19152, Electron Microscopy Sciences) for 1 h at 4°C, washed with cold-PBS, embedded in 2% low-melting agarose type II (#17856, Thermo Fisher Scientific, MA, USA) and dehydrated with cold ethanol series on ice for 2–5 min. Final alcohol dehydration was done twice using acetone-anhydride for 5 min and samples were infiltrated using Epon EMbed-812 resin (#14120, Electron Microscopy Sciences) at ratios 1:2, 1:1, and 2:1 with acetone for 5–30 min. After final infiltration the samples were polymerized at +60°C for 72 h. Using ultramicrotome (Leica EM UC7, Leica Microsystems, Germany) the samples were cut to 80 nm thick sections, collected onto copper-grids (Electron Microscopy Sciences), post-contrasted with 4% aqueous uranyl acetate (#2400, Electron Microscopy Sciences) for 1 h at RT and carbon-coated. Prepared samples were imaged at 120 kV using transmission electron microscope Jeol JEM 2100-Plus by the TEM center imaging software (Imaging Methods Core Facility, BIOCEV, Vestec, Czech Republic).

#### 2.4.2 Scanning electron microscopy (SEM)

For SEM, the samples were primarily fixed as described for TEM method. After three washing steps (15 min each) in buffer, the cells were fixed by buffered 1% osmium tetroxide for 1 h at 4°C and dehydrated with cold ethanol series (80–90–96%) for 2 min at RT each step. Ethanol was then replaced 1:1 with cold acetone for 5 min and finally, the anhydrous acetone was applied for 5 min before placing the samples into a Critical Point Dryer device (Leica EM CPD300). Samples were mounted on aluminum SEM stubs using carbon tape and sputter-coated in high-vacuum coater (Leica EM ACE600) with 4 nm of platinum. High-resolution images were obtained at accelerated voltage 1 kV and 0.1 nA using TLD detector operated in SE mode (FEI Helios NanoLab 660 G3 UC) (Imaging Methods Core Facility, BIOCEV, Vestec, Czech Republic).

#### 2.4.3 Electron microscopy image analysis

Fiji software (Schindelin et al., 2012) was used for quantification of the gold particles number per length of planar and microvillar membrane from TEM images. The average value was manually calculated from 10 cropped images of immunolabelled oocyte, where number (n) of particles per microvillar/planar membrane length (L) measured in nm was counted. With exclusion of the questionable areas below the *microvilli*, differences in the (n) of particles per microvillar/planar membrane length were analyzed using Mann–Whitney test (GraphPad Prism 9.3.1). *p*-value equal or lower 0.05 was considered to be significant (*p* ≤ 0.05*; 0.01**; 0.001***).

### 2.5 Proximity ligation assay

To detect the close proximity of proteins Juno and CD9, Proximity Ligation Assay (PLA)–Duolink *In Situ* Red Starter Kit, DUO92101 (Sigma-Aldrich^®^) was used as was described previously (Vondrakova et al., 2022). Fixed zona-free mouse mature oocytes (MII) were incubated overnight in 4°C with selected pair of primary antibodies: 1) experimental group: rat monoclonal anti CD9 (KMC8.8) (sc18869, Santa Cruz Biotechnology, Inc.) diluted 1:50 in 1% BSA and rabbit polyclonal anti Folate receptor 4 (Juno) (abx102438, Abbexa^®^, UK) diluted 1:50 in 1% BSA; 2) positive control–proteins with known interaction: mouse monoclonal anti-α tubulin diluted 1:1000 in 1% BSA (TU02) (sc8035, Santa Cruz Biotechnology) and rabbit polyclonal anti-β tubulin diluted 1:1000 in 1% BSA (ab15568, Abcam, UK); 3) negative control–protein with known absence of interaction: anti-α tubulin and rat monoclonal anti-CD9 (KMC8.8) diluted 1:50 in 1% BSA. Washed oocytes were incubated with PLUS and MINUS PLA probes and amplified following the manufacturer’s protocol and transferred into 2 μL of VECTASHIELD Mounting Medium. Fluorescence was detected with a confocal microscope (Carl Zeiss LSM 880 NLO) (Imaging Methods Core Facility, BIOCEV, Vestec, Czech Republic).

### 2.6 Detection of Juno and CD9 protein complex in HEK293T/17 cell line

#### 2.6.1 Co-transfection of HEK293T/17 cells

HEK293T/17 cells were seeded 24 h before transfection on cover slips placed in 60 mm-cultivation dishes. When cells reached 70–80% of confluence, they were co-transfected by 3 µg of mouse CD9-GFP plasmid DNA (MG226288, Origene, MD, USA) and 3 µg of mouse Juno plasmid DNA (MC207552, Origene) and left for 16 h in DMEM cultivation medium with 10% of FBS without antibiotics. Alternatively, HEK293T/17 cells were transfected only by 3 µg of mouse Juno plasmid DNA for control immunoprecipitation. The fluorescent signal of CD9-GFP transfected cells were visualized under the fluorescent microscope (Olympus CKX41, Olympus Corporation, Japan) using 20× objective. Cells were washed in ice-cold PBS buffer three times and lysed in non-reducing (2x) SDS (sodium dodecyl sulfate) lysis buffer for WB; and in lysis buffer, 1% CHAPS (Sigma-Aldrich^®^) in 30 mM Tris-HCl (pH 7.5) for co-immunoprecipitation. The sample without transfection (p−/−) was used as a negative control.

#### 2.6.2 Co-immunoprecipitation and Western blotting (WB) for Juno detection

Co-transfected/Juno transfected cell lysates were centrifuged 15,000 × g for 15 min 4°C, supernatants were incubated with magnetic beads (Dynabeads Protein G Immunoprecipitation kit, 10003D, Invitrogen, MA, USA) conjugated with rabbit polyclonal anti-GFP primary antibody (ab290, Abcam) overnight at 4°C on a rocking platform. Precipitated protein complex was eluted from immunobeads by elution buffer of Dynabeads kit (Dynabeads Protein G Immunoprecipitation kit, 10003D, Invitrogen, MA, USA), and then incubated with the reducing sample buffer for 5 min at 70°C. Protein lysates for WB were quantified using a NanoDrop 3000 spectrophotometer (Thermo Fisher Scientific, MA USA). Firstly, electrophoretic separation in 10% polyacrylamide gel was performed. Molecular mass was estimated with Prestained Precision Plus Protein Dual Color Standards (Bio-Rad, CA, USA). Tris-glycine buffer (pH 9.6) with 20% methanol was used for transfer of proteins onto a polyvinylidene difluoride (PVDF) membrane (Immobilon-P, Millipore, Germany). The electroblotting carried out for 1 h at 500 mA. PVDF membrane was blocked with 5% dry milk (Bio-Rad) in PBS-Tween. The targeted Juno protein from co-transfected (pJuno/pCD9-GFP) cell lysate, Juno transfected cells (pJuno/–), anti-GFP precipitate (pJuno/–) and precipitated Juno-CD9 complex (pJuno/pCD9-GFP) was detected by rabbit polyclonal anti-mFOLR4 (Juno) antibody (abx102438, Abbexa) diluted 1:500 in 5% low-fat milk. For control detection, rabbit polyclonal anti-GFP primary antibody in lysate from Juno transfected cells (pJuno/–) was used. After washing and incubation with secondary anti-rabbit IgG antibody conjugated with horseradish peroxidase (Bio-Rad) diluted 1:3000 in 5% milk, the protein signal was visualized using the SuperSignal Chemiluminescence Substrate (ThermoFisher Scientific) by the Azure c600 imaging system (Azure Biosystems, Inc., CA, USA).

#### 2.6.3 Mass spectrometry (MS) analysis

Co-immunoprecipitates were digested on the beads by trypsin. After O/N digestion at 37°C, samples were analyzed using a liquid chromatography system Agilent 1200 (Agilent Technologies) connected to the timsToF Pro PASEF mass spectrometer equipped with Captive spray (Bruker Daltonics). Mass spectrometer was operated in a positive data-dependent mode. Five microliters of peptide mixture were injected by autosampler on the C18 trap column (UHPLC Fully Porous Polar C18 0.3 × 20 mm, Phenomenex). After 5 min of trapping at a flow rate of 20 μL/min, peptides were eluted from the trap column and separated on a C18 column (Luna Omega 3 μm Polar C18 100 Å, 150 × 0.3 mm, Phenomenex) by a linear 35 min water-acetonitrile gradient from 5% (v/v) to 35% (v/v) acetonitrile at a flow rate of 4 μL/min. The trap and analytical columns were both heated to 50°C. Parameters from the standard proteomics PASEF method were used to set timsTOF Pro. The target intensity per individual PASEF precursor was set to 6000, and the intensity threshold was set to 1500. The scan range was set between 0.6 and 1.6 V s/cm^2^ with a ramp time of 100 ms. The number of PASEF MS/MS scans was 10. Precursor ions in the m/z range between 100 and 1700 with charge states ≥2+ and ≤6+ were selected for fragmentation. The active exclusion was enabled for 0.4 min.

The raw data were processed by PeaksStudio 10.0 software (Bioinformatics Solutions, Canada). The search parameters were set as follows: enzyme–trypsin (specific), carbamidomethylation as a fixed modification, oxidation of methionine and acetylation of protein N-terminus as variable modifications. The data were search against the *Mus musculus* database (Uniprot 12/2022).

### 2.7 CD9 structure prediction with homology modeling and structural comparison

Freely available MODELLER 9.25 software and python-based advanced scripting was used for generating the 3D structure of mouse CD9. Comparative structure prediction is divided into four steps. 1) Fold assignment for comparative modelling: one or more templates with a known sequence and 3D structure were identified for the modelling initiation. For known structure identification and assignment the BLASTp and Protein Data Bank curation was used. In the final stage of this fold assignment, a step profile was built by using build profile python scripting based on our query protein fasta sequence. 2) The sequence structure alignment: a target CD9 structure was aligned with the previously identified template structure with the help of another python script. This script reads the template PDB structures and the CD9 target sequence and performs the alignment; in the output, two different files “.ali” and “.pap” are generated. 3) Model python building: a script uses MODELLER’s auto model class was used to generate models, and each model was assessed with the normalized discrete optimized protein energy (DOPE) assessment method. 4) Evaluation: based on energy profile with the best DOPE score generated models were evaluated by using energy profile python script (Webb and Sali, 2016). The generated structures side chain was corrected with the help of the WHAT IF server (Vriend, 1990) and the quality of the structure was assessed using the PROCHECK server (Laskowski et al., 1993). This work used FASTA sequence of mouse CD9 that was retrieved from the NCBI data bank with accession number AAH70474. The used protein templates for MODELLER were, 6K4J, 5TCX, 6RLR, 6Z1V. The predicted structure was compared with AlphaFold predicted CD9 structure (accession number P40240). Furthermore, the MODELLER predicted structure was compared with Human CD9 (PDB id 6K4J) structure to assess their probable structural and functional similarity.

### 2.8 Protein-membrane modeling, protein-protein docking, visualization, and analysis

The protein-membrane model was prepared using CHARMM-GUI Membrane Builder online module (Lee et al., 2019). The protein-protein docking for mouse CD9-Juno, mouse Izumo1-Juno protein-protein interaction pose prediction was carried out using the ClusPro server (Kozakov et al., 2017) and interaction site prediction, analysis, and visualisation was carried out in Chimera 1.16. In this study protein crystal structures were depicted from RCSB Protein Data Bank, for mouse Juno crystal structure ID number 5JYJ, for mouse Izumo1 crystal structure ID number 5B5K, human Izumo1-Juno complex crystal structure ID number 5F4E were used.

All structures were visualized and hydrogen bonding between protein-protein structures were analyzed using a graphical user interference (GUI) based Chimera program.

### 2.9 Molecular dynamics simulation

Molecular Dynamics Simulations were used to understand the conformational motion of protein over time at atomic level. Furthermore, MD simulation processes help to understand protein motion over the time as well as their conformational changes to help interpret protein function. The MD simulation process is divided into several steps followed by protein topology formation, simulation box formation, and solvent addition (water in this case); ion addition for neutralizing the protein charge; energy minimization using energy minimization MD run in the steepest descent method; equilibration through position restraints and ensembles, and a final MD simulation run.

In this study MD simulations were performed at 300 K using the GROMACS 2020.1 software package in an Ubuntu Linux system by using an OPLS-All Atom force field. The whole system was packed in a cubic water box with a dimension of 10 Å by using the GROMACS gmx editconf module to set up the boundary conditions and GROMACS gmx solvate module for solvation.

Furthermore, the simulation systems were then packed in a simulation box using a SPC216 water model. For neutralizing the simulation system Na^+^ and Cl^−^ ions were added in the system box and the physiological system was maintained (0.15 M) using the GROMACS gmx genion module. The steepest descents method was used for energy minimization. The maximum step size along a 0.01 nm gradient with a maximum of 50,000 steps. Furthermore, the simulation system was equilibrated at a constant temperature of 300 K (= ∼27°C), by using an NVT and 100 ps NPT ensemble process. Firstly, a modified Berendsen thermostat with no pressure coupling was applied for NVT (constant number of particles, volume and temperature) canonical ensemble, and then the Parinello–Rahman method pressure of 1 bar (P) was applied the NPT ensemble (constant number of particles, pressure, and temperature). The final simulations were performed for each system for 10 ns where a leap-frog integrator was applied to the trajectories time evolution of the simulation trajectories (Sur et al., 2022). A series of simulation processes were performed for MODELLER predicted CD9, AlphaFold depicted CD9, Juno, and CD9-Juno.

## 3 Results

### 3.1 The interaction of Juno and CD9 within oolemma

**FIGURE 1:**
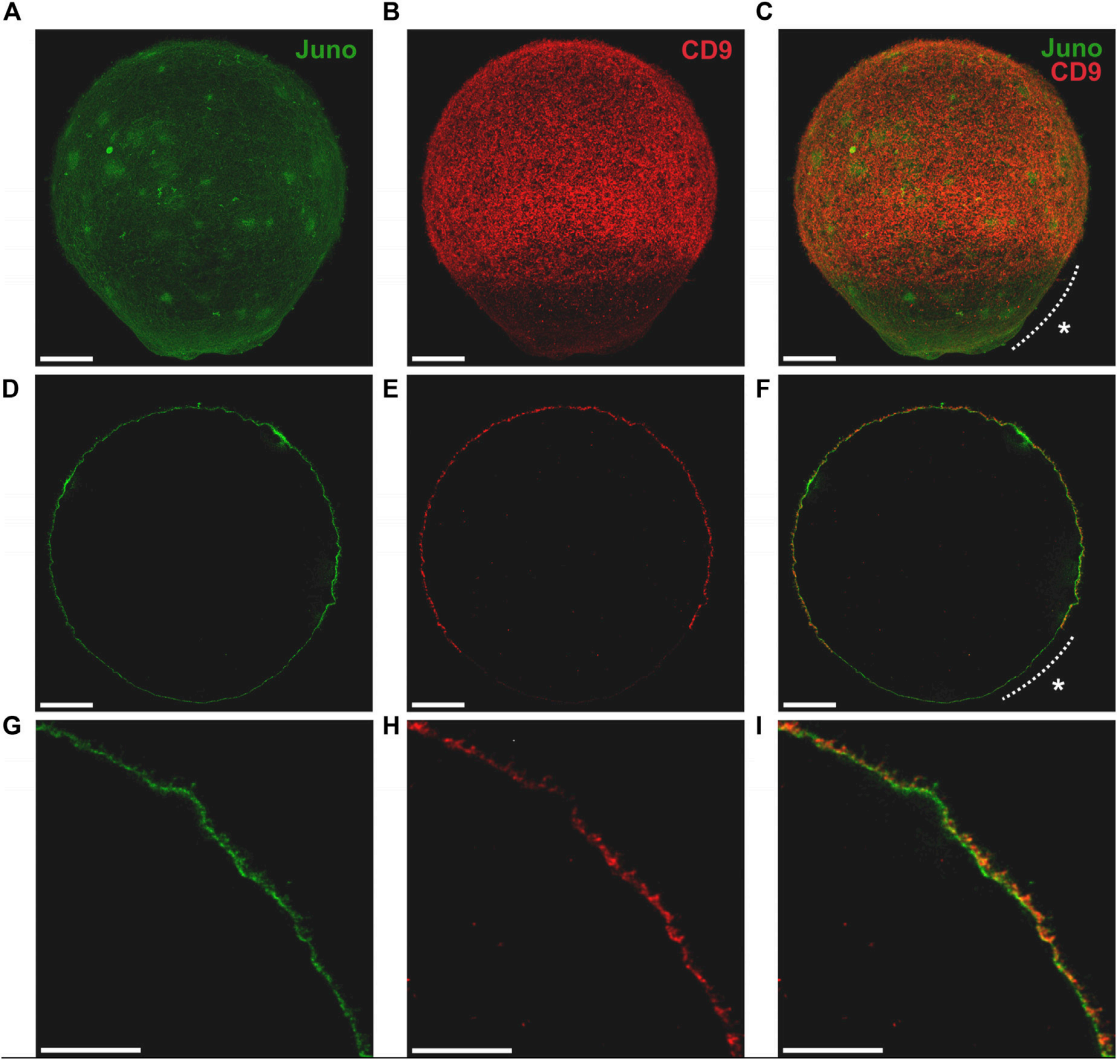
Visualization of Juno and CD9 localization in oolemma captured by 3D STED. Imaging of Juno (green) and CD9 (red) in oolemma **(A–C)** in whole oocyte surface visualized by maximal intensity projection, **(D–F)** in one plane and **(G–I)** in selected area of one plane. The asterisk (*) indicates *microvilli*-free region. Scale bar represents 10 μm **(A–F)**, 5 μm (**G–I)**. For more details see Supplementary Video S1.

**FIGURE 2:**
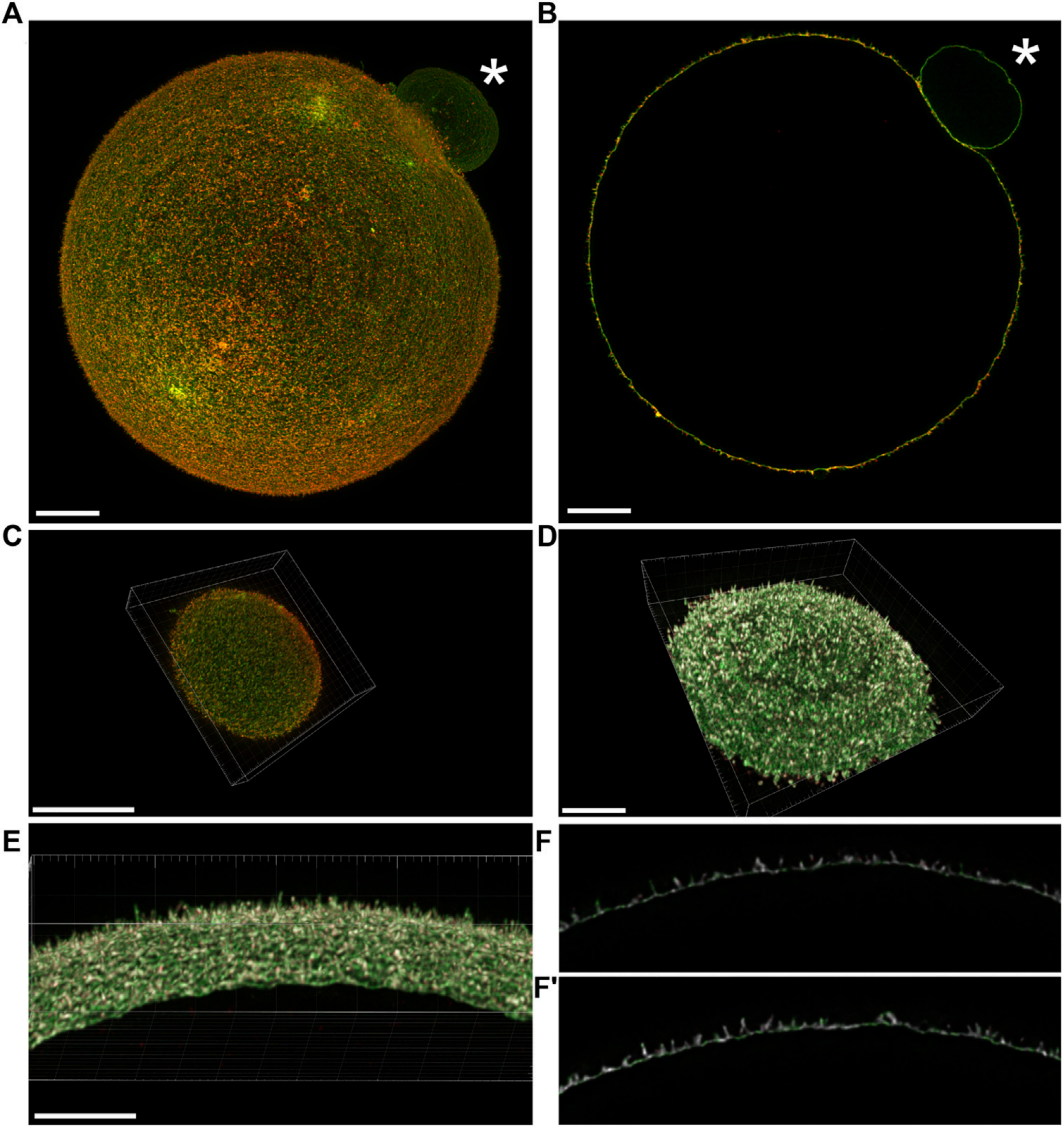
Visualization of the mutual position of Juno and CD9 in super-resolution images captured by 3D STED. **(A)** Imaging of Juno (green) and CD9 (red) in oolemma in whole oocyte surface visualized by maximal intensity projection and **(B)** in one plane. **(C)** A top and bottom segment of oocyte was captured for analysis of Juno and CD9 mutual localization within oolemma. **(D–F′)** The representative image analyzed by Imaris software shows the colocalization area (white) of the studied proteins in a top and bottom segment **(D)**, in selected area of oolemma **(E)** and in an individual plane **(F,F′)**. The asterisk (*) indicates polar body. Scale bar represents 10 μm **(A–D)**, 5 μm **(E)**. For details see Supplementary Videos S2, S3.

To address the mutual relationship of Juno and CD9 in oolemma of mouse oocyte, we first employed 3D STED super-resolution microscopy followed by colocalization analysis. In order to use 3D STED microscopy for capturing the entire volume of the oocyte, the optimization of the standard labelling procedure was necessary (Frolikova et al., 2023). The advanced protocol enabled us to achieve maximal lateral resolution near 70 nm and distinguished individual *microvilli* on oocyte surface. The optimization of the protocol resulted in the ability to capture the entire oocyte surface (Figure 1A–C; Figures 2A, B; Supplementary Videos S1, S2) and study the proteins of interest in the context of different oolemma compartments (Figures 1D–I; Figures 2C–F), namely, *microvilli* and *microvilli*-free regions across the whole oocyte measuring 80 μm in diameter. Our results of the entire oocyte surface imaging indicated that both Juno and CD9 proteins are located in the same surface structures within the *microvilli*-rich region of the oocyte (Figures 1A–C, Figure 2A). Using our antibodies, we also detected a low protein expression of Juno within the *microvilli*-free region in comparison to CD9 which was absent (Figures 1A–F). We performed a control by imaging oocytes labeled with antibodies against individual proteins using 3D STED (Supplementary Figure S1), to ensure that the localization of detected proteins was not influenced by artifacts. The results confirmed that the dual immunofluorescence labelling did not modify detected protein localization. Also, in the absence of primary antibodies, there was no detected signal given by secondary antibodies (Supplementary Figure S2).

In order to analyze in detail Juno-CD9 protein localization within oolemma *microvilli* (Figures 2A, B), we captured two segments of 2.4 μm in thickness (Figure 2C) as described in (Frolikova et al., 2023). In our study, we employ the terminologies “top” and “bottom” for these segments, to designate distinct sides of the oocyte. Specifically, the term “bottom” denotes the region of the oocyte that is situated in closest proximity to the microscope objective and the coverslip, while the term “top” refers to the opposing side of the oocyte, which is farthest from the coverslip. It should be noted that these designations are exclusively employed to convey the orientation of the oocyte relative to the microscope objective, and do not pertain to any inherent characteristics of the oocyte. These 3D STED obtained data were used for follow-up image analysis. The algorithm in MATLAB software (see 2. Materials and Methods) was developed for the evaluation of Juno and CD9 mutual position and the average value of Pearson’s correlation coefficient was measured for 8 oocytes (a top and bottom segment for 7 oocytes and only bottom for 1 oocyte were analyzed). The resulting average value of Pearson’s correlation coefficient was 0.67 ± 0.06 indicating mutual colocalization of the proteins of our interest. Based on Pearson’s correlation coefficient between Juno and CD9 labeling, the representative colocalization map was prepared using Imaris software (Figures 2C–F, F′; Supplementary Video S2). The results of this analysis provided evidence for the mutual proximity and potential existence of Juno and CD9 association.

**FIGURE 3:**
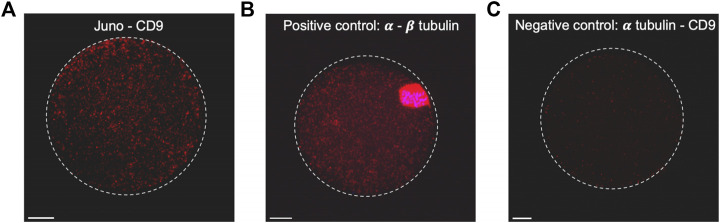
Study of close proximity of Juno and CD9 in mouse oocyte oolemma by PLA. **(A)** The presence of positive signal (red dots) on the sample stained by Juno and CD9 and visualized by maximal intensity projection, confirmed the existence of their close proximity, **(B)** α and β tubulin-stained sample was used as a positive control of the method. **(C)** Combination of α tubulin and CD9 staining was used as a negative control. Chromosomes are visualized with Dapi (blue). Scale bar represents 10 μm.

Based on results of the colocalization analysis, the existence of Juno and CD9 association was subsequently investigated. PLA (Alam, 2018) was employed to detect association of Juno and CD9 in mouse oolemma (Figure 3). The PLA demonstrated a robust dotted signal in an experimental group of oocytes when primary antibodies against Juno and CD9 were used (Figure 3A; Supplementary Figures S3). As a positive control the interaction between α and β tubulin was used and the strongest signal was detected in the peri-chromosomal region. In contrast, hardly any signal was detected in a group considered as a negative control, when primary antibodies against α tubulin and CD9 were used, as these two proteins are not predicted to interact (Figure 3C). Given that PLA has been previously reported as a highly sensitive and specific powerful method for detecting protein-protein interactions *in situ* (Alam, 2018), and based on our results, we conclude to detect a potential interaction between Juno and CD9.

**FIGURE 4:**
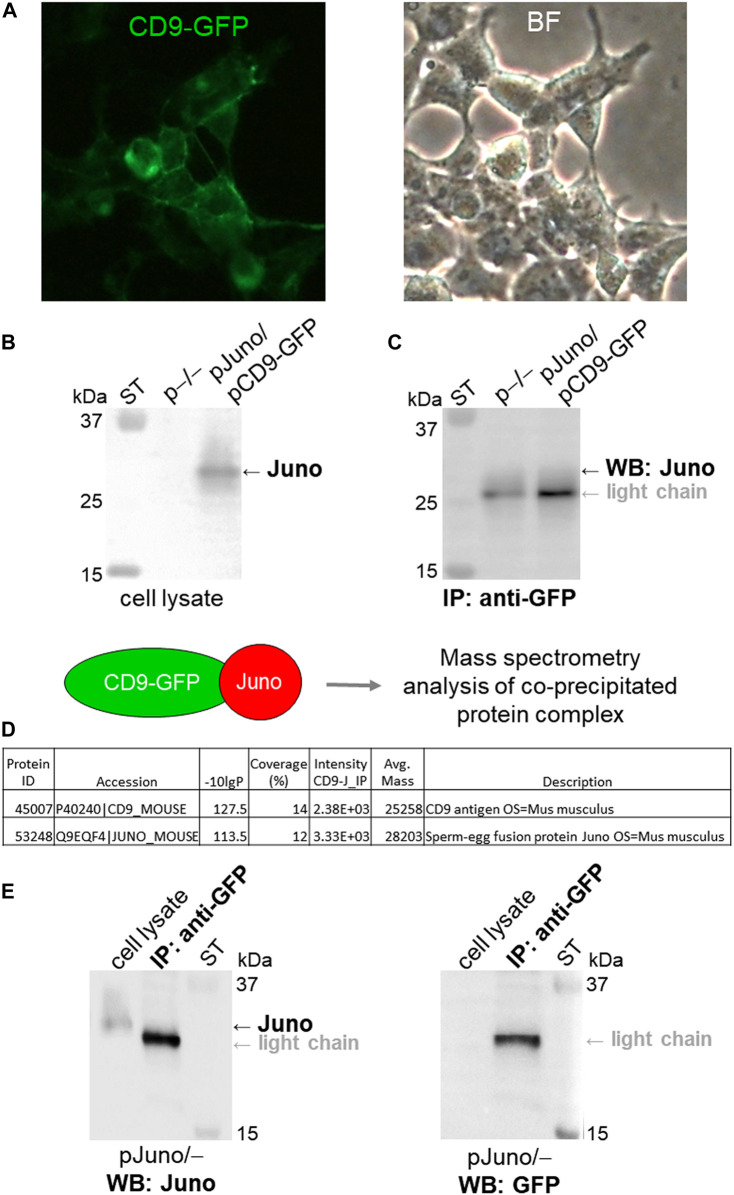
Analysis of Juno-CD9 protein-protein interaction via co-immunoprecipitation and MS. **(A)** HEK293T/17 cells were co-transfected with Juno and CD9-GFP mouse plasmids (pJuno/pCD9-GFP). CD9 protein was visualized in the cell membrane immediately after transfection via fluorescent GFP-tag (green); BF (bright field). **(B)** Juno protein expression was confirmed by WB and visualized by anti-Juno antibody. **(C)** Juno-CD9 complex was precipitated via GFP-tag on CD9 using anti-GFP antibody. CD9-bound target protein Juno was detected by using WB analysis with anti-Juno antibody (∼28 kDa); ST–molecular standards. **(D)** Schematic figure depicting MS analysis of the protein complex, which was bound to immunobeads. Database-search algorithms (bioinformatics) were used to identify specific proteins based on their mass spectra; see raw data files Table 1 MS_IP; Table 2 MS_IP control; https://biobox.biocev.org/index.php/s/4NCxYE2ckAJ4eCP). For both WB and MS the non-transfected HEK293T/17 cells were used as a negative control (p−/−), and neither CD9 nor Juno was detected and identified. **(E)** As a control for GFP-tag antibody specificity, the Juno and GFP antibody detection was performed in the anti-GFP immunoprecipitate (IP) and in Juno transfected cell lysate (pJuno/–), respectively; ST–molecular standards.

To further address Juno-CD9 interactions the HEK293T/17 cells were co-transfected by a mouse Juno plasmid and the CD9 plasmid with a GFP-tag for protein visualization (Figure 4A) and mouse Juno protein expression in transfected HEK293T/17 cells was confirmed by immunodetection with antibody in cell lysate (Figure 4B). The Juno-CD9 complex was precipitated via GFP-tag on CD9 using anti-GFP antibody. Although the Juno detection was partially masked by the light chain of the antibody used for immunoprecipitation, Juno antibody labeled the band of approximately 28 kDa, which was not visible in the non-transfected (p−/−) cell lysate (Figure 4C). For confirmation, the Juno-CD9 complex bound on the immunobeads was subjected to MS analysis (Figure 4D, raw data files Table 1 MS_IP; Table 2 MS_IP control; https://biobox.biocev.org/index.php/s/4NCxYE2ckAJ4eCP), which confirmed the presence of both proteins. GFP-tag immunoprecipitation specificity was confirmed by investigation of Juno protein in the anti-GFP immunoprecipitate and negative detection of Juno protein in Juno transfected (pJuno/–) cell lysate with GFP antibody (Figure 4E).

### 3.2 Nanoscale resolution of Juno and CD9 localization in *microvilli* rich compartments

Although Juno and CD9 was previously reported to be present in oolemma (Kaji et al., 2000; Le Naour et al., 2000; Miyado et al., 2000; Bianchi et al., 2014) the accurate localization of Juno in distinct compartments of the oolemma has not been identified. It is crucial to understand, that the membrane, which forms the *microvilli*-rich region consists of two distinct compartments, such as microvillar membrane (MvM) and planar membrane between individual *microvilli* (PlnM).

**FIGURE 5:**
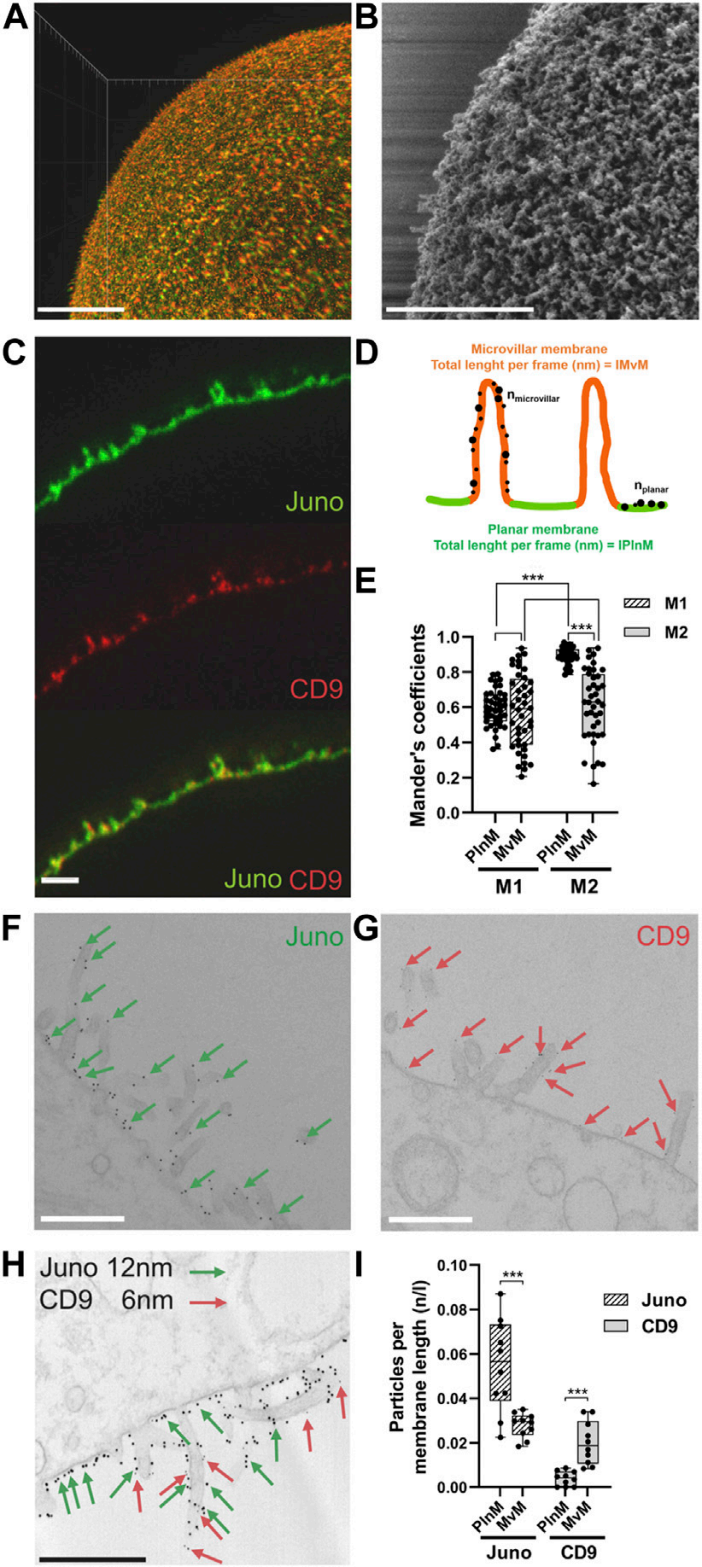
Localization of Juno and CD9 in MvM and PlnM compartments of *microvilli*-rich oolemma. **(A)** STED microscopy visualization of *microvilli* **(B)** SEM visualization of *microvilli*. **(C)** STED imaging revealed differences in Juno (green) and CD9 (red) localization within MvM and PlnM compartments of oolemma. **(D)** Scheme represents dividing the individual oolemma compartments for image analyzis of STED and TEM data. **(E)** Comparison of the localization of Juno and CD9 in MvM and PlnM separately in the segmented regions of oolemma shows significant difference between Meander’s coefficient 1 (M1) and Meander’s coefficient 2 (M2) in PlnM and significant difference in Meander’s coefficient 2 (M2) between MvM and PlnM regions. **(F–H)** Juno and CD9 differences captured by TEM, Juno (green arrows) was present in both MvM and PlnM in contrast to CD9 (red arrows) which was mainly detected in MvM. **(I)** TEM image analysis confirmed significant differences in localization Juno and CD9 both between MvM and PlnM compartments. Scale bar represents 10 μm **(A)**, 1 μm **(C)** 500 nm **(B,F–H)**. *p*-value equal or lower 0.05 was considered to be significant (*p* ≤ 0.001***). n–number of golden particles, l–total length per frame (nm), n/l–number of particles on total length per frame.

We combined indirect immunodetection captured by 3D STED super-resolution microscopy and electron microscopy (Figure 5) to determine mutual localization of Juno and CD9 in different *microvilli*-rich membrane compartments in nanoscale resolution. Firstly, 3D STED super-resolution microscopy for imaging of the whole oocyte surface enabled us to visualize the oolemma in such fine details (approximately 70 nm) that *microvilli* can be distinguished (Figure 5A) and is comparable to SEM (Figure 5B). While Juno-corresponding homogenous signal was localized in both MvM and PlnM compartments, CD9 localization was concentrated mainly in MvM (Figure 5C). For evaluation and quantification of these differences in Juno and CD9 localization, image analysis based on Manders’ correlation coefficients was used. The individual parts of the membrane used for analysis are illustrated in the scheme presented in Figure 5D. The average value of Manders’ correlation coefficient was measured for 8 oocytes (a top and bottom segment for 7 oocytes and only bottom for 1 oocyte were analyzed). The resulting value for coefficient M1 corresponding to Juno signal over CD9 signal overlap was 0.68 ± 0.13, while the value for coefficient M2 corresponding to CD9 signal over Juno signal overlap was 0.82 ± 0.10. These results could be interpreted that CD9 has the same localization as Juno more frequently, than Juno with CD9 and indirectly confirmed the 3D STED imaging-based observation. To compare the localization of Juno and CD9 in MvM and PlnM separately, we analyzed the Manders’ coefficients in the segmented regions of the oolemma sections. The differences in M1 and M2 in both regions were quantified (Figure 5E). There was no significant difference in M1 when comparing PlnM *versus* MvM, however, there was a significant difference (*p* ≤ 0.001) in M2 between these two regions. Also, the resulting value of coefficient M1 showed a statistically significant difference from M2 in PlnM (*p* ≤ 0.001), indicating that CD9 has the same localization as Juno more frequently than *vice versa*, while showing no significant difference between the coefficients in MvM.

These findings correspond with the results from transmission electron microscopy (TEM), where the data showed that Juno is localized in both oolemma compartments (MvM and PlnM), while CD9 is preferentially localized in MvM. Subsequently, the differences in microvillar membrane compartment localization of Juno and CD9, visualized by TEM (Figures 5F–H) were quantified (Figure 5I). The quantification showed statistically significant differences in expression of studied protein between MvM and PlnM. Data showed that higher expression of Juno was detected in PlnM compared to MvM (*p* ≤ 0.001) and confirmed the higher expression of CD9 (*p* ≤ 0.001) in MvM compared to PlnM.

The results suggest that although there exist differences in expression of Juno and CD9 between MvM and PlnM compartments, both proteins are expressed in the MvM part of oolemma. This finding suggests that Juno-CD9 could assist during primary sperm-oocyte binding in MvM prior to gamete membrane fusion.

### 3.3 CD9 structure prediction with homology modeling

The mouse Juno crystal structure was obtained from the RCSB protein data bank. Due to the unavailability of the mouse CD9 crystal structure, the CD9 structure was predicted using a python algorithm-based MODELLER. The primary amino acid sequence of mouse CD9 was used for BLASTp analysis and close PDB hit finding. The BLASTp PDB search showed that mouse CD9 has the highest sequence similarity with human CD9 protein (PDB id 6K4J), and human tetraspanin CD81 (PDB id 5TCX). The best matched PDB structures were selected as a template for mouse CD9 3D structure prediction through homology modelling. Homology modelling with MODELLER was used to prepare several 3D models of mouse CD9. Based on their lowest DOPE score (−25904.08203) the best fit model was selected (Supplementary Figure S4). The model was additionally used for missing side chain building and quality assessment through the What If and PROCHECK servers. The PROCHECK server assessment allowed us to achieve an overall structural quality of 92%, and a Ramachandran plot (Supplementary Figure S5). The results of a comparison study between the predicted structures of CD9 by MODELLER and AlphaFold indicate a high degree of structural similarity, with a calculated similarity score of 98.17% (Supplementary Figure S6). The results of a comparison study between the structures of mouse CD9 and human CD9 (PDB id 6K4J) showed a high degree of sequence identity between these proteins, specifically 87.61% of their amino acid sequences are identical. Additionally, a Blast search revealed that mouse CD9 possesses 89.04% amino acid sequence identity with 100% query coverage (Supplementary Figures S7, S8).

### 3.4 Protein-membrane modeling, protein-protein docking, visualization and analysis

**FIGURE 6:**
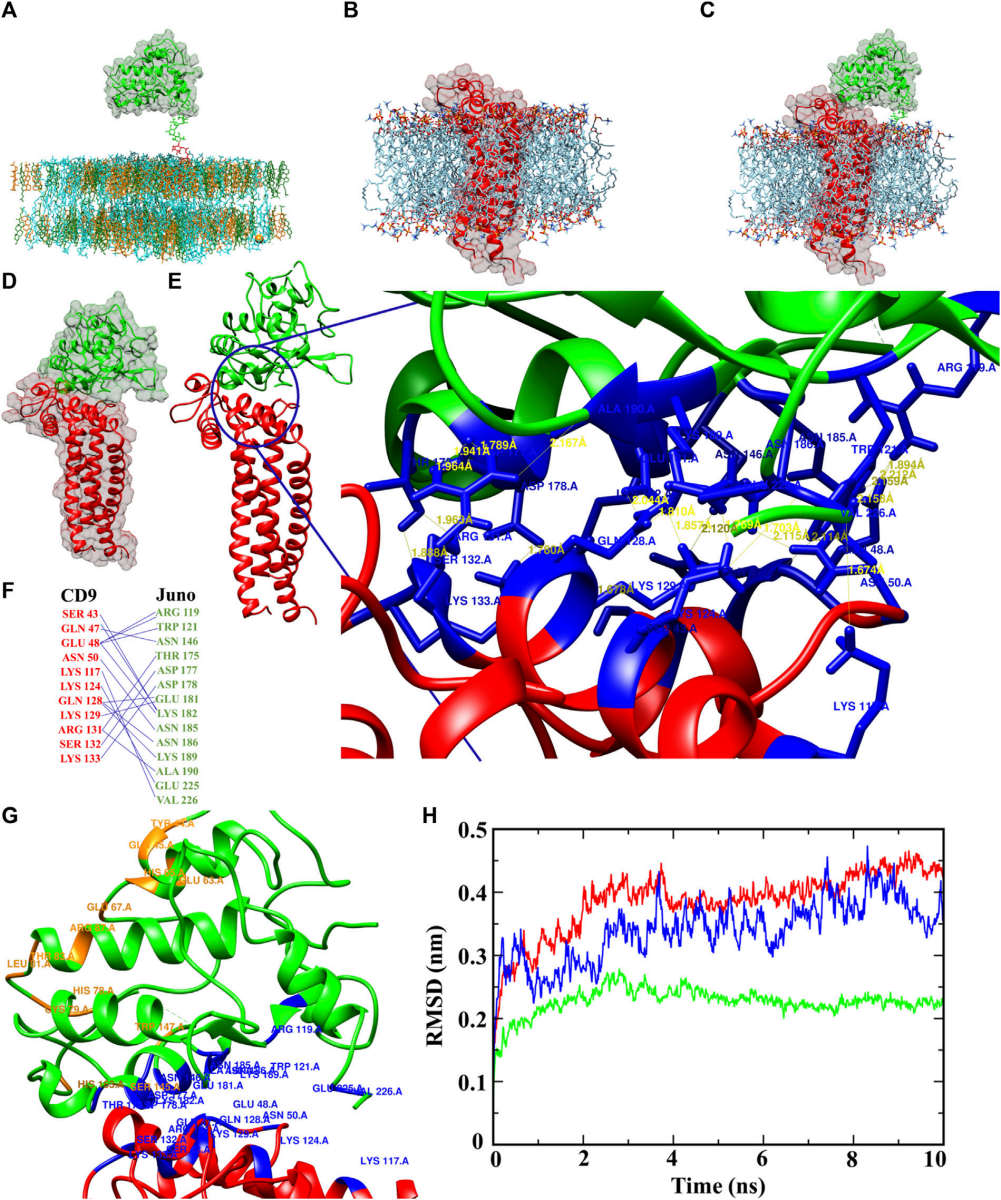
Juno-CD9 interactive model within the oocyte oolemma. **(A–C)** Biologically relevant position of Juno and CD9, shown by interacting pose in the membrane model for **(A)** Juno, **(B)** CD9, **(C)** Juno-CD9 prepared in CHARMM-GUI. **(D)** Energetically adequate and biologically most favorable and significant docked pose of Juno-CD9. **(E)** Interacting amino acids of Juno and CD9, where 26 hydrogen bonds were formed between Juno and CD9, and the distances between donor hydrogen and acceptor can be seen. **(F)** Responsible amino acids from Juno and CD9 responsible for hydrogen bonds formation and Juno-CD9 complex formations. **(G)** Juno-CD9 docked pose, where blue marked residues from Juno and CD9 are interacting for making the complex, whereas the orange residues were identified (either by docking or human Izumo1-Juno crystal analysis) for interacting with Izumo1. **(H)** RMSD plot for Juno (green), CD9 (red), and Juno and CD9 complex (blue). Based on RMSD analysis, Juno maintain the highest level of stability around 2.2 Å, CD9 showed highest deviation around 5.5 Å, whereas Juno and CD9 complex displayed deviation, 3.6 Å, in the middle between CD9 and Juno readings. The Juno-CD9 complex was reflected by highly stable conformation during the MD simulation which indicated a stable biological relevant structural conformation of Juno and CD9 complex.

The protein-membrane model gives the relevant location of the protein in the membrane and position of the transmembrane regions. The protein-membrane model was prepared to visualize Juno and CD9 position within a membrane lipid bilayer (simulating oolemma) to recognize their possible interacting mode. GPI anchored Juno (Figure 6A), tetraspanin CD9 (Figure 6B), and Juno and CD9 potential interaction (Figure 6C) were predicted by a Dioleoyl phosphatidylcholine (DOPC) membrane model. The most favorable interaction complexes using the ClusPro server with a PIPER algorithm were ranked according to the lowest free energy and were grouped based on protein-protein interaction features. The scored best fit and biologically relevant structural position of Juno and CD9 docked model was visualized and analyzed by Chimera 1.16 (Figure 6D) where interaction of Juno in close proximity to CD9 was detected. Furthermore, the intramolecular hydrogen bonds between the protein molecules were analyzed. The docking analysis revealed 21 (Supplementary Table S1) specific hydrogen bond network formed between 14 amino acid residues of Juno and 11 amino acid residues of CD9 in the Juno-CD9 complexes. Intermolecular hydrogen bond analysis through the Chimera 1.16 platform revealed that most of the donor-acceptor distances were within the ideal distance parameters of 2.7–3.0 Å (Supplementary Table S1) (Harris and Mildvan, 1999). This indicated hydrogen bond formation and Juno-CD9 complex establishment. The bond distances (between a hydrogen donor and acceptor) indicated energetically favorable Juno and CD9 interacting mode (Figures 6E, F and Supplementary Table S1). Salt bridges could also play a role in proteins’ complex formation, however, docking analysis did not reveal the presence of salt bridges between the proteins of interest. This could be interpreted as, salt bridges do not play a role in the Juno-CD9 complex formation. This study has suggested that the amino acid residues responsible for Juno interaction with Izumo1 may remain free to interact with Izumo1 even during the Juno-CD9 complex. To explore this possibility, two different sets of approaches were taken.

The first approach involved docking of mouse Izumo1-Juno complexes, showing the probability of Juno residues responsible for the interaction with CD9 in mouse marked in red; residues in yellow from mouse Juno are responsible for the interaction with mouse Izumo1; residues in magenta from mouse Izumo1 are responsible for interaction with mouse Juno (Supplementary Figure S9A and Supplementary Table S2). Our findings predicted that the amino acid residues of mouse Juno responsible for interaction with CD9 and Izumo1 were distinct and did not interfere with each other (Supplementary Figure S9A and Supplementary Tables S1, S2). These findings provide further support for the hypothesis that the amino acid residues involved in mouse Juno-Izumo1 interaction are likely to remain open during the mouse Juno-CD9 complex and available to bind Izumo1 protein. Mouse Izumo1-Juno docked complex was found to involve six residues of Juno interacting with eight residues of Izumo1, forming a robust network of 11 hydrogen bonds (Supplementary Figure S9B and Supplementary Table S2).

Further, the comparison of the previously published human Juno-Izumo co-crystal structure (PDB id 5F4E) revealed 11 amino acid residues that are involved in the interaction between Juno and Izumo1, as shown in Supplementary Figure S10A. Importantly, the result of our docking study showed that five of these residues in mouse Juno involved in Izumo1 interaction were identical with the amino acid residues involved in Juno-Izumo1 binding interface in the human crystal structure (Supplementary Figure S10B marked in yellow). These five residues were shown to remain empty during mouse Juno-CD9 interaction and could be predicted to remain available for mouse Juno-Izumo1 binding. These findings may suggest that specific amino acid residues in mouse Juno may play a critical role in binding to Izumo1 and provide further evidence to support the hypothesis that these residues are not involved in the Juno-CD9 interaction. Our data showed that all the amino acid residues in mouse Juno that could be involved in Izumo1 binding (based on either by docking or human Juno-Izumo1 interaction analysis, marked orange) did not overlap with the residues interacting with mouse CD9 (Supplementary Figure S10, marked red). These findings are supported by results showing that the amino acid residues responsible for the interaction of mouse Juno with Izumo1 are likely not involved in the mouse Juno-CD9 interaction (Figure 6G and Supplementary Table S1) and remain free. Specifically, docking and predicted interaction data suggest that mouse Juno-Izumo1 interacting interface contains residues (TYR 44, GLN 45, GLU 63, GLU 67, ARG 87, HIS 163, HIS 65, CYS 79, LEU 81, THR 83, SER 145, TRP 147) that are different from those of the Juno-CD9 interacting side (ARG 119, TRP 121, ASN 146, THR 175, ASP 178, ASP 177, GLU 181, LYS 182, ASN 185, ASN 186, LYS 189, GLU 225, VAL 226).

### 3.5 Molecular dynamics simulation

To analyze experimentally obtained data from the wet lab, we performed computational MD simulation to study the docked Juno-CD9 protein complex, the stability, compactness, and various physicochemical parameters such as, flexibility, structural deviation, and energy level. Root mean square deviation (RMSD) of backbone atoms were calculated over the entire 10 ns simulation trajectories, where RMSD values for Juno, CD9, and Juno-CD9, are ∼2.2 Å, ∼4.3 Å, ∼3.6 Å, respectively (Figure 6H), also on in reference the CD9 structure from AlphaFold showed same RMSD ∼4.3 Å value (Supplementary Figure S11). All RMSD values indicated that all the protein structures including the predicted and docked Juno-CD9 interacting structure are in a stable biologically relevant condition which is in accordance with the obtained microscopical data. The structural stability data from MD simulation of Juno and CD9 also confirmed highly stable Juno and CD9 complex. Secondary structure analysis showed Juno contains 32% alpha helical structure, 6.1% beta strands, and 61.9% coiled structure with no turns found. Post MD analysis showed no changes in the secondary structure of Juno (Supplementary Figure S12). In the case of CD9, 77.4% alpha helical structure, 7.1% turns, and 15.5% coiled structure but no beta strands were found. After simulation CD9 protein showed a striking change in secondary structure with, 76.1% alpha helical structure and 23.9% coiled structure but interestingly, CD9 protein lost all the turns by that stage (Supplementary Figure S13). The complex of Juno-CD9 displayed 54.80% alpha helical structure, 4.3% beta strands, 10.2% turns, and 30.7% coiled, whereas post MD analysis showed 53.2% alpha helical structure, 3.3% beta strands, and 43.5% coiled and no turns were found (Supplementary Figure S14). It is clear that coil structure plays important roles in protein-protein interaction (Grigoryan and Keating, 2008). Therefore, our results support the Juno-CD9 complex formation, as both Juno and CD9 contain coil structures and extended coils are formed during the interaction. Further, during formation of Juno-CD9 complex there are larger numbers of coil structures which indicate their uninterrupted interaction over the 10 ns MD simulation time. As MD simulation represents a reflection of biological process, it could be predicted that in a biological system Juno and CD9 interact through their coil-coil structures, which is supported by presented microscopic data (Figure 3). Flexibility is an important phenomenon especially for protein-protein interaction (Grünberg et al., 2006). Based on our data, Juno did not change to any coils after the MD simulation run which could be interpreted as a non-flexible biological behavior of Juno. In contrast, when Juno is in interacting mode with CD9, the complex undergoes secondary structural changes indicating that structural flexibility is an important factor of protein-protein side chain interaction. The pre and post MD simulation data of secondary structure analysis clearly indicated that Juno-CD9 complex undergoes structural changes, which is in confirmation with the phenomenon of protein-protein interaction (Tobi and Bahar, 2005). With the knowledge that flexibility is also important for protein-protein interaction (Demirel and Keskin, 2005) the root mean square fluctuation (RMSF) technique was applied to evaluate protein flexibility (Sur et al., 2022). Atomic range RMSF analysis (Supplementary Figure S15) indicates that the Juno, CD9 and the complex of Juno-CD9 fluctuates in a stable range except the c-terminal atoms of the Juno-CD9 complex, where Juno showed rigid patterns in the atomic range. On the other hand, CD9 displayed a higher level of flexibility than Juno, and the Juno-CD9 complex mimicked the fluctuation pattern from both individual Juno and CD9 proteins. In the atomic range fluctuation study, it could be seen that Juno and CD9 exerted a fluctuation pattern mostly like CD9 with a higher flexibility. These results support the prediction of complex formation, where CD9 extends the side chains and contributes to the Juno-CD9 complex formation more than Juno, which agrees with the secondary structure data analysis (Supplementary Figures S12–S14). Along with other parameters, an electrostatic force which also plays a crucial role in protein-protein binding (Shashikala et al., 2019) was measured and an energy profile of Juno, CD9 and Juno-CD9 were obtained. Based on the results, in the case of Juno-CD9 complex formation, a short range electrostatic Coulombic energy seems to play a crucial role. Specifically, Coulombic energy for Juno was very low (88.3046 kg/mol) in contrast to the high energy for CD9 (−349.1170 kg/mol), with even higher readings for the Juno-CD9 complex (−727.8620 kg/mol). From the Coulombic energy profiling it could be deducted that the CD9 molecule contributes the highest energy towards the complex formation and the complex gains a higher electrostatic force during Juno-CD9 interaction resulting in a largely stable structure (Brock et al., 2007). There was no significant result found of short-range Lennard–Jones interaction (Supplementary Figure S16). Furthermore, the protein enthalpy plays an important role in protein-protein interactions, as it determines the overall energy change during the interaction. A favourable enthalpy change indicates that the interaction is energetically favourable and contributes to the stability of the complex. The protein enthalpy was measured as −188.69 kJ/mol (Juno), −410.06 kJ/mol (CD9), and −862.06 kJ/mol (Juno-CD9). The significantly more negative enthalpy for the Juno-CD9 complex suggests a stronger interaction between Juno and CD9 compared to their individual molecules and understanding the underlying mechanisms of protein-protein interactions (Bogan and Thorn, 1998) (Supplementary Figure S16). The Gibbs free energy for Juno (−2.06 kJ/mol), CD9 (−2.69 kJ/mol, and Juno-CD9 (−6.92 kJ/mol) further indicated the formation of a strong and stable condition for the thermodynamic reaction for Juno-CD9 complex formation (Supplementary Figure S17). Importantly, hydrogen bonds (H-bonds) are also crucial for protein stability, interaction, and protein folding (Erijman et al., 2014). Based on MD simulation H-bond analysis, it was predicted that during Juno-CD9 interaction possesses 278 sidechain H-bonds, in contrast to individual molecules of Juno (113 H-bonds) or CD9 (126 H-bonds). The H-bond numbers during the simulation period indicate that the Juno-CD9 complex gained 39 hydrogen bonds (Supplementary Figure S18). Based on protein-solvent interaction the Juno-CD9 complex possesses 808 H-bonds, in contrast to Juno (455 H-bonds) or CD9 (434 H-bonds). These H-bond numbers indicate that the Juno-CD9 complex loses 81 H-bonds towards the solution, and they could be possibly involved in Juno-CD9 complex. This interpretation most probably explains why these 81 H-bonds were not available for protein-solvent interaction and clearly indicates that the Juno-CD9 complex forms a higher hydrophobic core (Supplementary Figure S19). MD Simulation was captured as a movie file for better understanding of the dynamic state of Juno, CD9 Juno-CD9, and AlphaFold CD9 (Supplementary Videos S4–S7).

## 4 Discussion

Although, Juno and CD9 are the only oocyte surface presented proteins confirmed to be essential for mouse fertilization (Siu et al., 2021), their mutual relationship is unclear. Published data suggested the existence of Juno and CD9 cooperation within oolemma (Jégou et al., 2011; Inoue et al., 2020; Mori et al., 2021) strengthen by their protein expression in the same membrane compartments (Inoue et al., 2020; Mori et al., 2021). Using colocalization assay we detected Juno and CD9 colocalization in the *microvilli*-rich part of mouse oolemma which was absent in the *microvilli*-free region, which further supports previous findings (Runge et al., 2007; Inoue et al., 2020).

In relation to the detail of our study, we herein considered the existence of distinct compartments within mouse oolemma surface and possibility of spatially regulated structures (Mori et al., 2021), which are predicted to play a role in sperm-oocyte binding and could be determined by Juno and CD9 localization. The aspect of protein density, that could determine the Juno-CD9 close proximity and have an impact on oolemma microdomain function needed to be tested. Previously, STED microscopy was employed to analyze the microvillar region of the mouse oocyte (Wang and Kinsey, 2022). This study by Wang and Kinsey (2022) focused on imaging of the sperm-binding site, therefore, a relatively small area of oolemma was imaged. In contrast, using our newly developed strategy with a modified protocol for 3D STED super-resolution microscopy (Frolikova et al., 2023), we captured the entire oolemma with a high resolution of around 70 nm, which is unique for such large objects as oocytes, and it represents a significant improvement of previously used methods (Frolikova et al., 2023). This enabled us to distinguish two oolemma compartments within the *microvilli*-rich region. The findings that Juno protein expression is significantly elevated in planar membrane between individual *microvilli* while CD9 localization is confined to be mainly in microvillar membrane provides the evidence, that only certain parts of oolemma may likely serve as sperm binding regions. We could speculate that the existence of significant differences in Juno and CD9 distribution between planar and microvillar membrane regions has a functional reason, such as regulation of sperm attachment in terms of sperm number and consequential fusion of gamete membranes. The individual differences in Juno and CD9 oolemma location stress their different individual roles particularly for Juno in the blocking of polyspermy (Bianchi et al., 2014) and for CD9 in maintaining membrane curvature (Frolikova et al., 2018). Sperm fusion is likely facilitated in presence of CD9, which is localized in the membrane of extracellular vesicles (Miyado et al., 2008) as well as exclusion of Juno from *microvilli*-free region (Inoue et al., 2020) by RanGTP signaling, which may regulate localization of Juno through CD9 protein (Mori et al., 2021). However, their mutual localization and comparable protein amount in MvM points to the functional importance of Juno-CD9 complex formation for primary sperm-oocyte membrane recognition. This could be initiated by Juno-CD9 interface pocket in the microvillar part of oolemma surface supported by protein-membrane position modelling, with biologically relevant membrane orientation including Juno GPI anchor, and CD9 transmembrane domains (Umeda et al., 2020).

Furthermore, protein-protein flexible side chain docking depicted the most energetically and biologically relevant interacting formation reflected by an elevated number of hydrogen bonds that give stability to the Juno-CD9 complex. As proteins are dynamic structures within living biological systems, we addressed their activity by MD simulation followed by flexible side chain docking and discovered the most suitable interacting modes based on stability and fluctuation pattern. The docking pose and the higher stability of Juno-CD9 interacting modes and energy scale (mostly Gibbs free energy) supported experimentally derived Juno and CD9 complex. Mutual interaction of Juno with CD9 within the plasma membrane was confirmed by co-immunoprecipitation followed by MS identification of both proteins. Additionally, the GFP antibody used for co-IP did not react with Juno protein, indicating a specific pull-down of the Juno-CD9 complex via the GFP-tag from co-transfected cells.

Juno is shaded from oolemma in extracellular vesicles derived from the oolemma *microvilli*-rich part (Bianchi et al., 2014), and CD9 is present in the vesicles within the oocyte perivitelline space (Miyado et al., 2008). Based on these findings, we propose that the interaction between Juno and CD9 proteins in oolemma of MII oocyte, may precede or assist to Juno-Izumo1 binding during early stages of fertilization. This finding is supported by the knowledge that tetraspanins, such as CD9, are part of extracellular vesicles formation and their cargo (Andreu and Yáñez-Mó, 2014). The fact that both proteins are part of the extracellular vesicles released from the oocyte surface suggested their interaction not only before, but also after sperm-oocyte binding and membrane fusion including a formation and release of extracellular vesicles of oolemma origin that likely assist in block of polyspermy (Bianchi et al., 2014). The possible interaction between Juno and CD9 is further supported by the presented structural study, which revealed that CD9 protein possesses a high degree of sequence identity, with 87.61% structural similarity between mouse and human. Furthermore, a Blast search analysis revealed that Mouse CD9 shares 89.04% amino acid sequence identity with 100% query coverage. These findings support the theory that CD9 could play a similar role in fertilization in both mouse and human, and the structural features and functional properties of CD9 are conserved throughout evolution.

In summary, based on analysis of multiple disciplinary derived data, we propose the existence of Juno-CD9 complex in specific spatially defined compartments of microvillar regions of mouse oolemma with a distinct functional role in sperm-oocyte binding. Furthermore, these Juno-CD9 complexes may serve as competent microdomains for sperm-oocyte fusion.

## Data Availability

Source data files corresponding to individual panels in Figures 1–6 are deposited in as Source Data Archives 1-6. Link: https://biobox.biocev.org/index.php/s/4NCxYE2ckAJ4eCP.
