# Supplementary material for: Juno and CD9 protein network organization in oolemma of mouse oocyte
Source: Front Cell Dev Biol. 2023 Aug 10;11:1110681. doi: 10.3389/fcell.2023.1110681 (PMC10450504; doi:10.3389/fcell.2023.1110681)
Supplement: Supplementary file 3 [file DataSheet1.pdf]

Supplementary file for

Juno and CD9 Protein Network Organization in Oolemma of Mouse Oocyte

Michaela Frolikova[1], Vishma Pratap Sur[1], Ivan Novotny[2], Michaela Blazikova[2], Jana Vondrakova[1], Ondrej Simonik[1], Lukas Ded[1], Eliska Valaskova[1], Lenka Koptasikova[3], Ales Benda[3], Pavla Postlerova[1,4], Ondrej Horvath[2], Katerina Komrskova[1,5*]

[1]Laboratory of Reproductive Biology, Institute of Biotechnology of the Czech Academy of Sciences, BIOCEV, Vestec, Czech Republic.

[2]Light Microscopy Core Facility, Institute of Molecular Genetics of the Czech Academy of Sciences, Prague, Czech Republic.

[3]Imaging Methods Core Facility at BIOCEV, Faculty of Science, Charles University, Vestec, Czech Republic.

[4]Department of Veterinary Sciences, Faculty of Agrobiology, Food and Natural Resources, University of Life Sciences Prague, Prague, Czech Republic.

[5]Department of Zoology, Faculty of Science, Charles University, BIOCEV, Prague, Czech Republic.

* Correspondence:
Katerina Komrskova, Ph.D.
katerina.komrskova@ibt.cas.cz

This file contains:

Supplementary Figures S1-S19

Supplementary Table S1 and S2

List of Supplementary Videos S1-S6

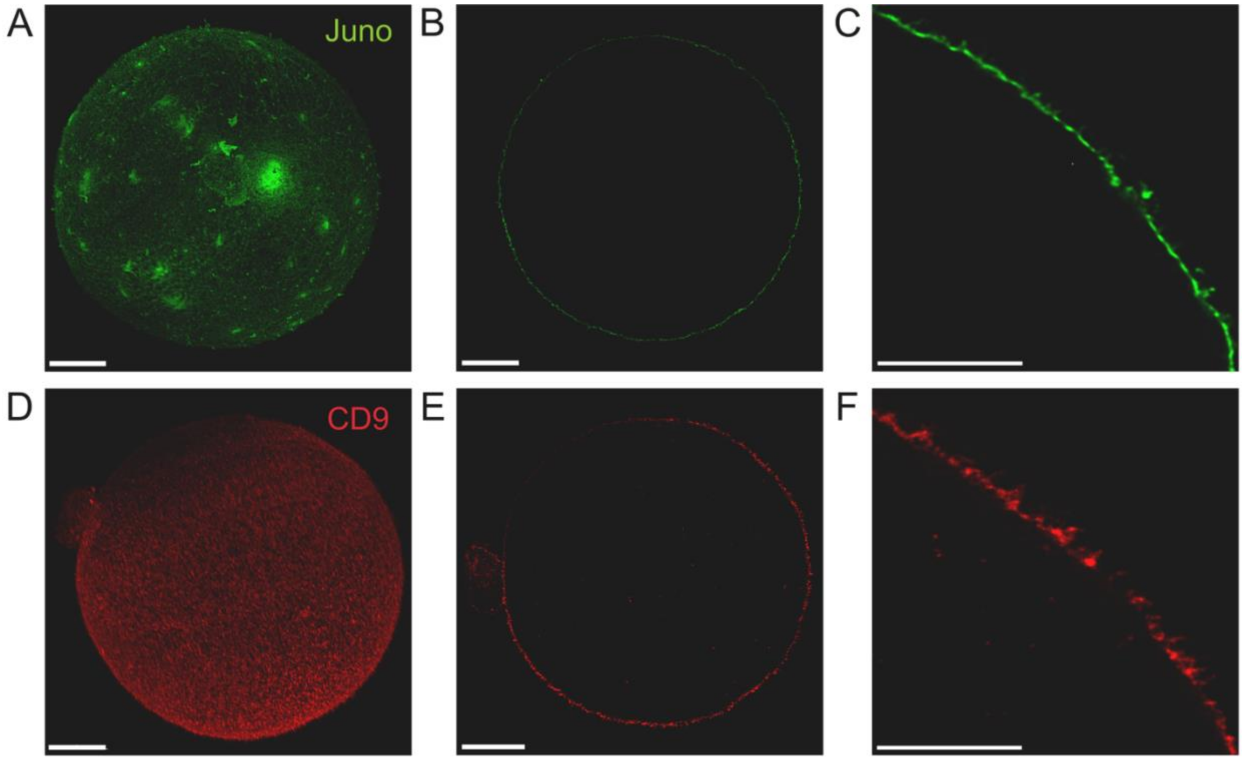

Supplementary Figure S1: Visualization of Juno and CD9 localization in oolemma captured by 3D STED. (**A-C**) Imaging of Juno (green) and (**D-F**) CD9 (red) in oolemma. (**A,D**) Localization of individual studied proteins in whole oocyte surface visualized by maximal intensity projection, (**B,E**) in one plane and (**C,F**) in selected area of one plane. Scale bar represents 10 mm (**A,B,D,E**), 5 mm (**C,F**).

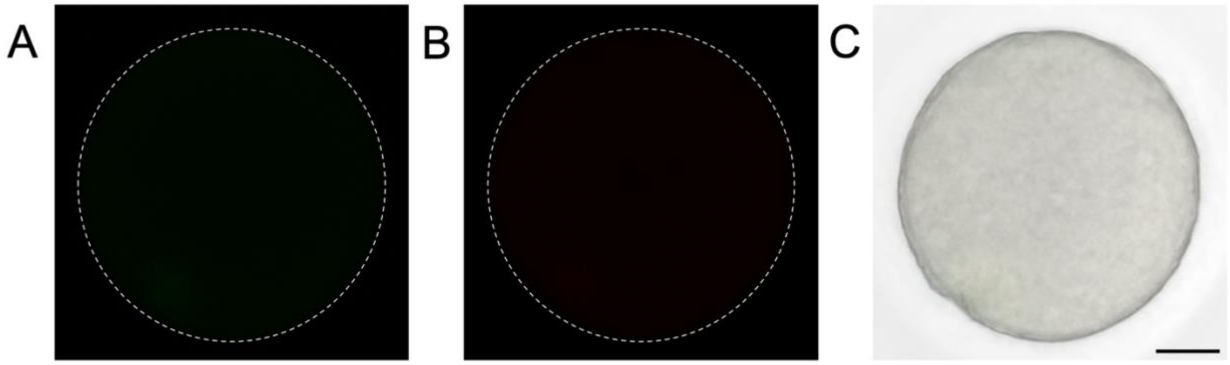

Supplementary Figure S2: Negative control for immunofluorescence staining. No signal was detected after using secondary antibodies (**A**) anti-rabbit IgG Abberior STAR 580 and (**B**) anti-rat IgG Abberior STAR 635P without using primary antibodies. (**C**) The oocyte is visualized with Bright-Field microscopy. Scale bar represents 10 mm.

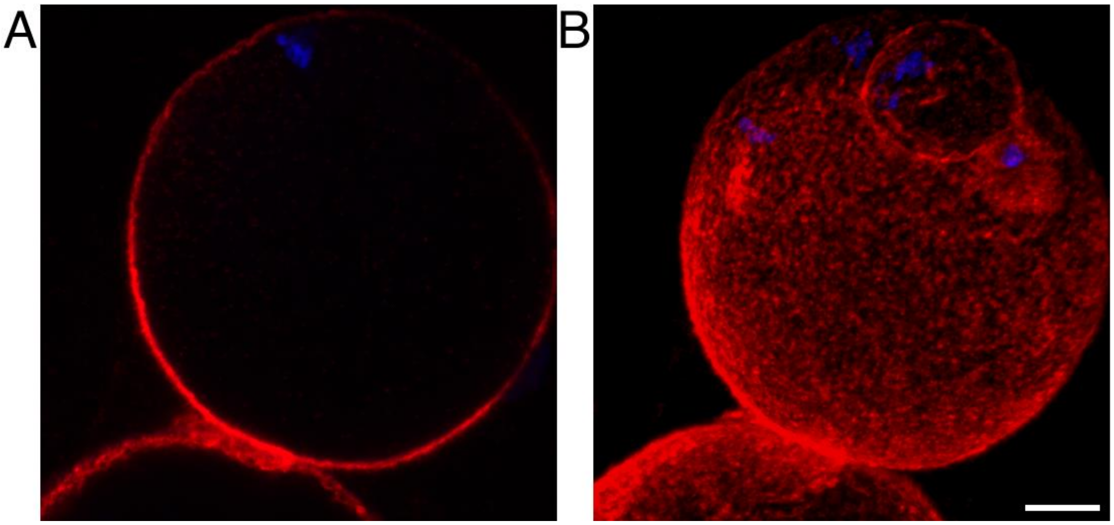

Supplementary Figure S3: Interaction of Juno and CD9 in mouse oocyte *oolemma* detected by PLA. The positive signal (red dots) of anti-Juno and anti-CD9 antibodies imaged (**A**) by one plane, and (**B**) by maximal intensity projection, detected the existence of Juno-CD9 mutual interaction. Chromosomes are visualized with Dapi (blue). Scale bar represents 10 μm.

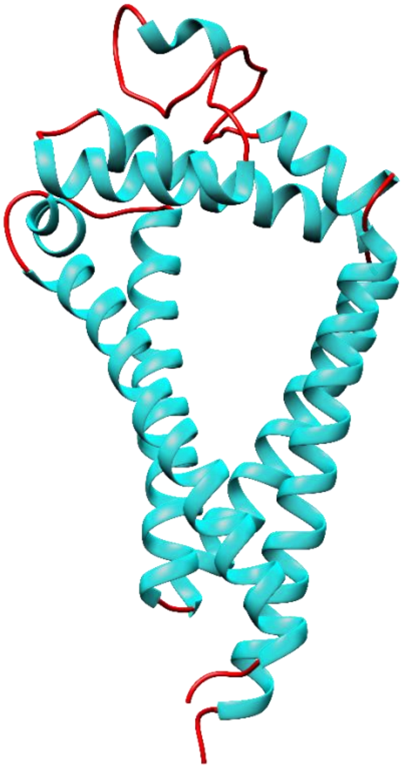

Supplementary Figure S4: Mouse CD9 structure predicted by advanced MODELLER package.

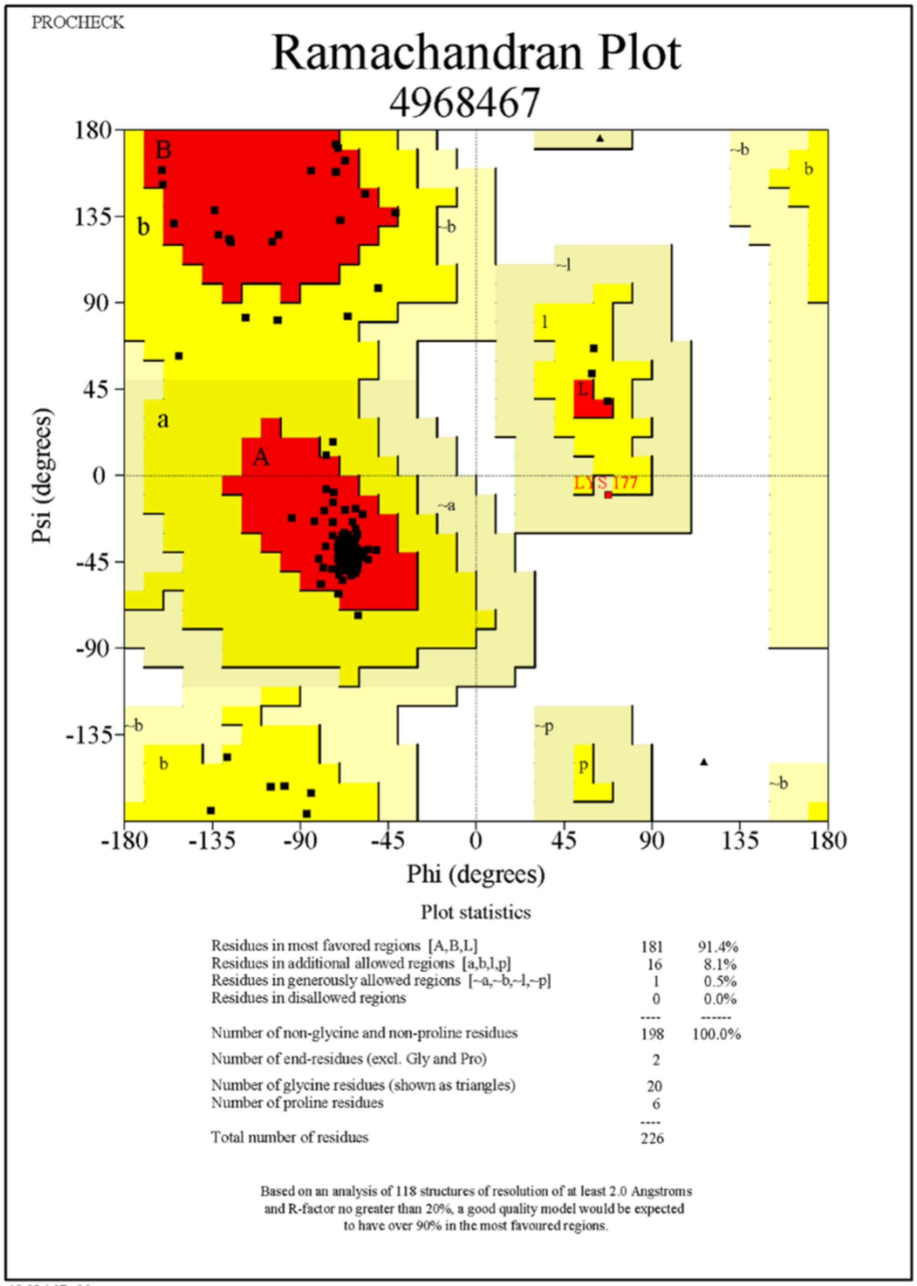

Supplementary Figure S5: Ramachandran Plot for the theoretical model of CD9 protein structure for quality assessment of the build structure. We can see that most of the residues are in the most favored regions, and we can assume that more than 90% of residues in the most favored region indicates a good quality structure comparable to crystal structure quality.

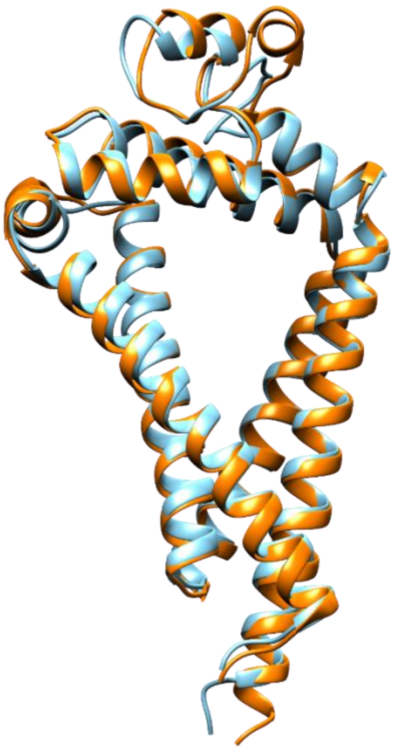

Supplementary Figure S6: Structural comparison between the mouse CD9 structure predicted by advanced MODELLER package (cyan) and CD9 predicted structure aquired from Alphafold server (orange), and Structural similarity found 98.17%.

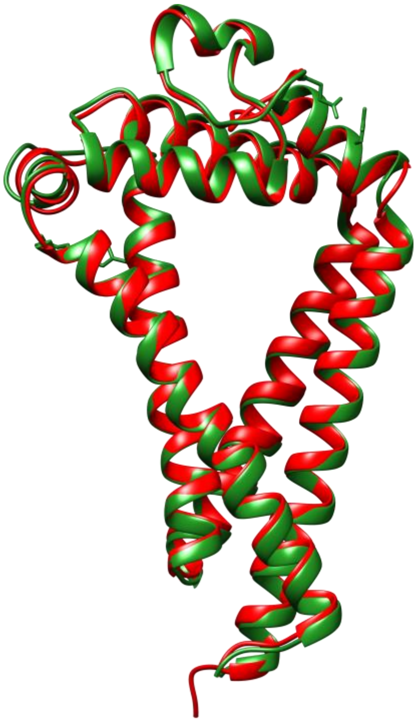

Supplementary Figure S7: Mouse CD9 and Human CD9 structural comparison where we found 87.61% structural similarities.

Download GenPept Graphics

CD9 antigen [Mus musculus]

Sequence ID: NP_031683.1 Length: 226 Number of Matches: 1

See 6 more title(s) See all Identical Proteins(IPG)

Range 1: 1 to 226 GenPept Graphics ▼ Next Match ▲ P

Score	Expect	Method	Identities	Positives	Gaps
420 bits(1079)	3e-156	Compositional matrix adjust.	203/228(89%)	218/228(95%)	2/228(0%)

```
Query  1    MPVKGGTKCIKYLLFGFNFIFWLAGIAVLAIGLWLRFDSQTKSIFEQETNNNNSSFYTGV  60
            MPVKGG+KCIKYLLFGFNFIFWLAGIAVLAIGLWLRFDSQTKSIFEQE  NN+SSFYTGV
Sbjct  1    MPVKGGSKCIKYLLFGFNFIFWLAGIAVLAIGLWLRFDSQTKSIFEQE--NNHSSFYTGV  58

Query  61   YILIGAGALMMLVGFLGCCGAVQESQCMLGLFFGFLLVIFAIEIAAAIWGYSHKDEVIKE  120
            YILIGAGALMMLVGFLGCCGAVQESQCMLGLFFGFLLVIFAIEIAAA+WGY+HKDEVIKE
Sbjct  59   YILIGAGALMMLVGFLGCCGAVQESQCMLGLFFGFLLVIFAIEIAAAVWGYTHKDEVIKE  118

Query  121  VQEFYKDTYNKLKTKDEPQRETLKAIHYALNCCGLAGGVEQFISDICPKKDVLETFTVKS  180
            +QEFYKDTY KL++KDEPQRETLKAIH AL+CCG+AG +EQFISD CPKK +LE+F VK
Sbjct  119  LQEFYKDTYQKLRSKDEPQRETLKAIHMALDCCGIAGPLEQFISDTCPKKQLLESFQVKP  178

Query  181  CPDAIKEVFDNKFHIIGAVGIGIAVVMIFGMIFSMILCCAIRRNREMV  228
            CP+AI EVF+NKFHIIGAVGIGIAVVMIFGMIFSMILCCAIRR+REMV
Sbjct  179  CPEAISEVFNNKFHIIGAVGIGIAVVMIFGMIFSMILCCAIRRSREMV  226
```

Supplementary Figure S8: Mouse CD9 and Human CD9 sequence blast revealed that both sequence shares 89.04% amino acid sequence identity with 100% query coverage.

Supplementary Table S1: List of Hydrogen bonding with donor – acceptor and distance.

H-Bond network		Donor	Acceptor	Donor hydrogen-Acceptor distance (Å)	Donor-Acceptor distance (Å)
CD9	Juno				
ASN 50	ASN 186	ASN 186	ASN 50	2.115	2.986
LYS 117	VAL 226	LYS 117	VAL 226	1.674	2.655
GLU 48	ARG 119	ARG 119	GLU 48	2.212	3.089
GLU 48	ARG 119	ARG 119	GLU 48	1.894	2.819
GLU 48	TRP 121	TRP 121	GLU 48	2.158	2.993
LYS 124	GLU 225	LYS 124	GLU 225	1.703	2.647
LYS 124	GLU 225	LYS 124	GLU 225	1.769	2.771
GLN 128	GLU 181	GLN 128	GLU 181	2.044	2.925
LYS 129	GLU 181	LYS 129	GLU 181	1.857	2.769
ARG 131	ASP 177	ARG 131	ASP 177	1.941	2.794
ARG 131	ASP 177	ARG 131	ASP 177	1.964	2.789
ARG 131	ASP 177	ARG 131	ASP 177	1.789	2.696
ARG 131	ALA 190	ARG 131	ALA 190	2.167	2.987
SER 132	THR 175	THR 175	SER 132	1.963	2.804
SER 132	THR 175	SER 132	THR 175	1.780	2.790
LYS 133	ASP 178	ASP 178	LYS 133	2.120	3.020
GLN 47	ASN 146	ASN 146	GLN 47	1.888	2.707
SER 43	LYS 182	LYS 182	SER 43	1.678	2.696
GLN 47	ASN 185	ASN 185	GLN 47	2.114	3.045
GLU 48	ASN 185	ASN 185	GLU 48	2.159	3.082
GLN 128	LYS 189	LYS 189	GLN 128	1.810	2.777

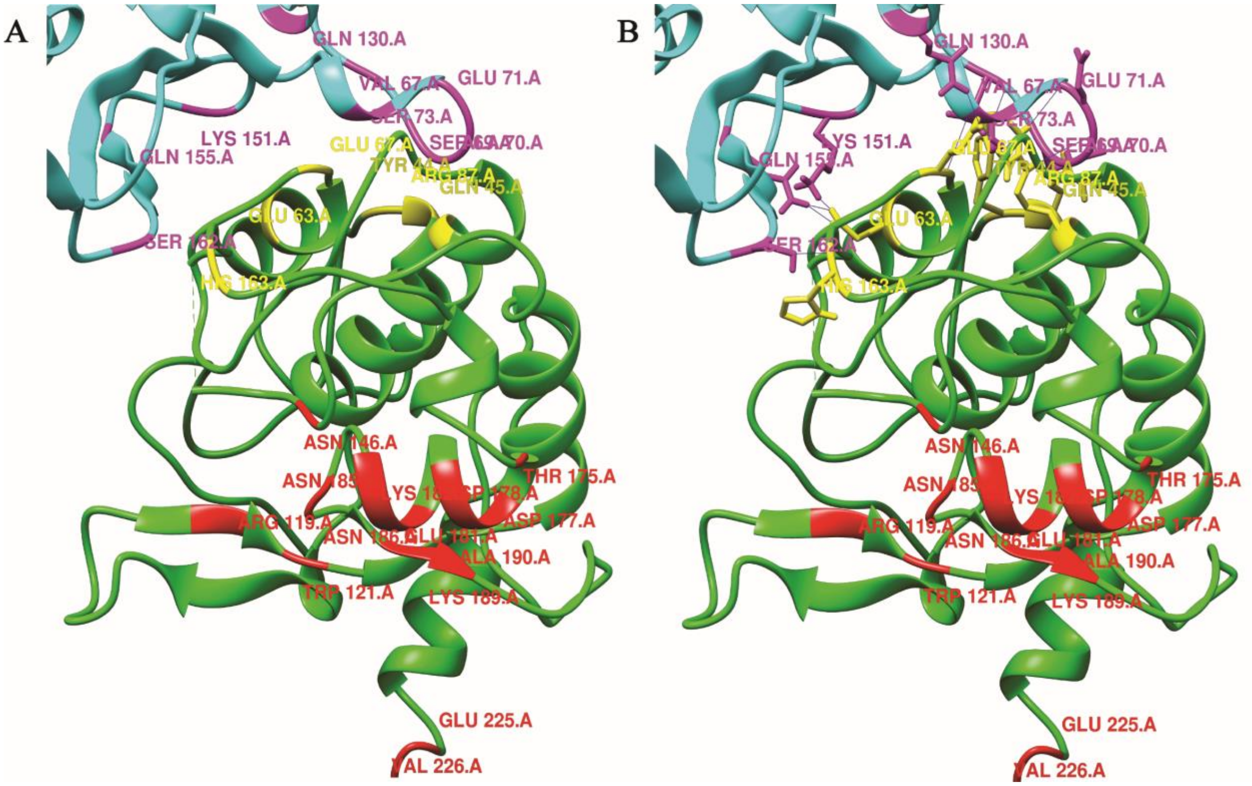

Supplementary Figure S9: Mouse Izumo1-Juno docked result with free amino acid residues for CD9 interaction. (**A**) Interacting mode of Mouse Juno (green) and Izumo1 (cyan) after docked in Cluspro 2.0 server and analyzed in Chimera, where residues marked yellow belongs to juno, residues marked with magenta belongs to Izumo1 were maninly responsible for mouse Izumo1-Juno interaction. Furthermore amino acid residues of mouse juno marked red was responsible for interatcion with mouse CD9, from here it is very clear that during mouse Juno-Izumo1 interaction the amino acids site needed for interaction with CD9 was remains open and available for interatcion with CD9 protein during Juno-Izumo1 interaction. (**B**) H-bonds between mouse Juno-Izumo1 can be seen when Juno was interacting with Izumo1 with free amino acids site for CD9 interaction.

Supplementary table S2: List of Hydrogen bonding in Izumo1-Juno inetaraction with residue details, donor – acceptor and distance.

H-Bond network		Donor	Acceptor	Donor hydrogen-Acceptor distance (Å)
Izumo1	Juno			
GLN 130	GLU 67	GLN 130	GLU 67	2.352
GLN 155	GLU 63	GLN 155	GLU 63	2.292
LYS 151	GLU 63	LYS 151	GLU 63	1.728
SER 73	GLU 67	SER 73	GLU 67	2.063
SER 69	GLN 45	SER 69	GLN 45	1.875
SER 73	GLU 67	SER 73	GLU 67	1.946
ALA 70	ARG 87	ARG 87	ALA 70	2.319
GLU 71	ARG 87	ARG 87	GLU 71	1.766
GLN 155	GLU 63	GLN 155	GLU 63	2.091
SER 162	HIS 163	HIS 163	SER 162	1.905
VAL 67	TYR 44	TYR 44	VAL 67	1.854

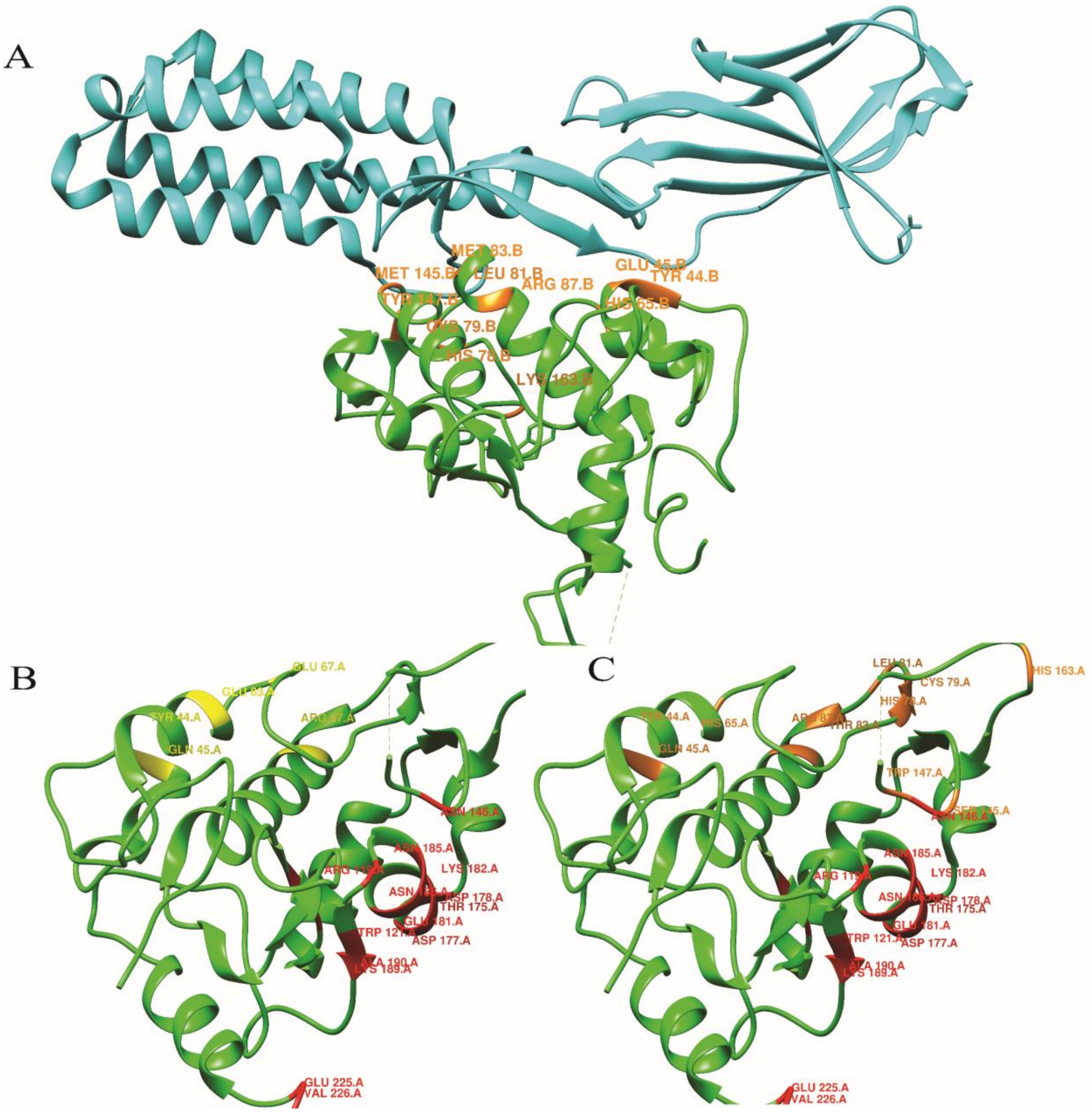

Supplementary Figure S10: Juno with highlighted residues participating in interaction with Izumo1 and CD9. (**A**) Human Juno-Izumo1 interatcing pose where interacting residues of Juno highlighted with orange colour. (**B**) Mouse Juno with highlighted residues with yellow colour is responsible for interacting with mouse Izumo1 (docked result, further discussed in the next figure), wheather residues marked red colour is responsible for interacting with CD9. As per both analysis amino acids of Juno is free and available for interation with Izumo1. (C) Mouse Juno with highlighted residues with orange colour might be responsible for interacting with mouse Izumo1 (as per human Juno-Izumo1 interaction (Ohto et al., 2016)), wheather residues marked red colour is responsible for interacting with CD9.

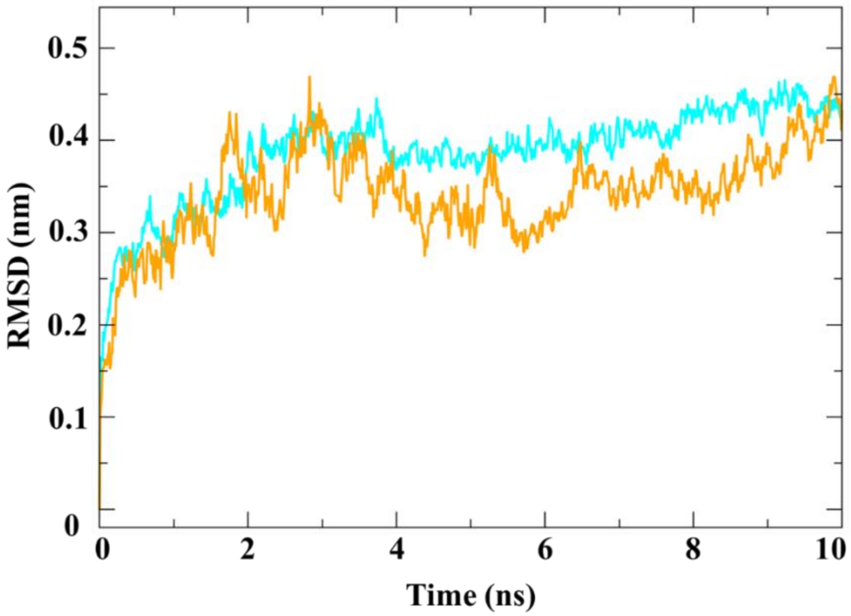

Supplementary Figure S11: RMSD comparison is indicating the MODELLER predicted CD9 (cyan) and Alphafold collected CD9 (orange) is sharing the almost same fluctuation until 3.5ns (4Å) and MODELLER predicted CD9 continue until end of the simulation and possess 4.3Å RMSD value whereas Alphafold CD9 fluctuate more than MODELLER CD9 structure but maintain an lower RMSD value (3.5 - 4Å) but at the end of simulation it also possess 4.3Å RMSD value with higher fluctuation rate than the MODELLER predicted CD9.

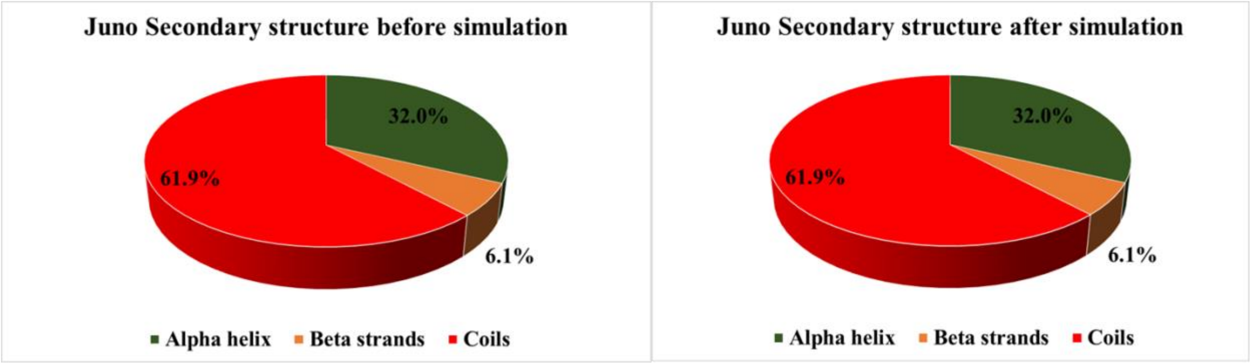

Supplementary Figure S12: The secondary structure of Juno before simulation and after simulation where no changes were found during the 10 ns molecular dynamics simulation period. Molecular dynamics of proteins reflect the biological process of protein folding unfolding protein-protein interaction, so we can assume that Juno exerts the same characteristics in the biological system.

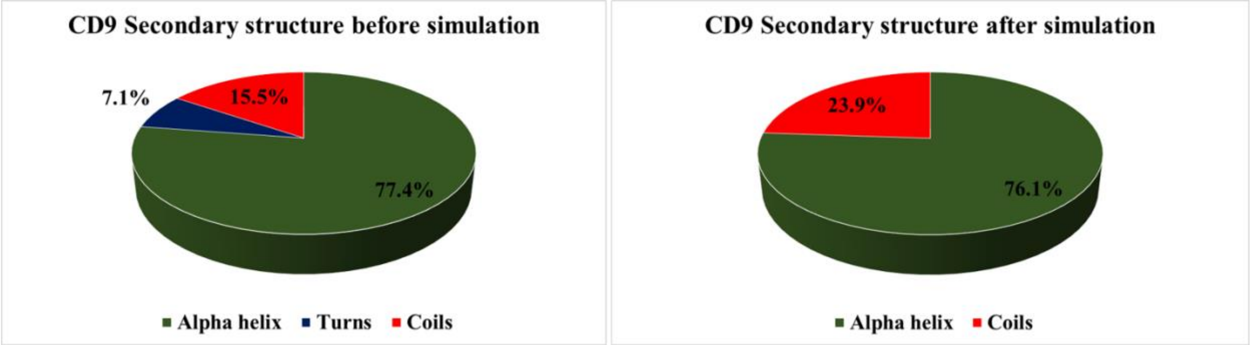

Supplementary Figure S13: The secondary structure of CD9 before simulation and after simulation, where we can see that CD9 protein went through a lot of structural changes during the 10 ns molecular dynamics simulation period. Molecular dynamics of proteins reflects the biological process of protein folding unfolding protein-protein interaction, so we can assume that CD9 exerts the same characteristics in the biological system.

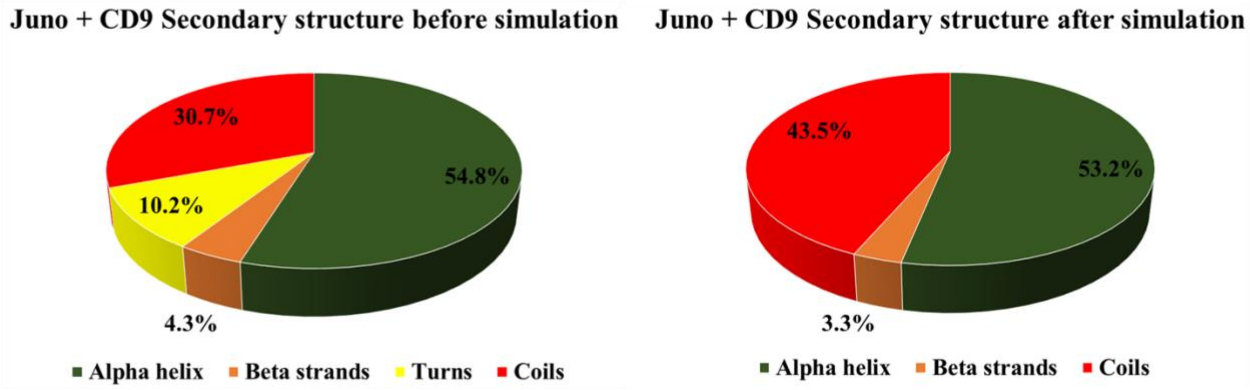

Supplementary Figure S14: The secondary structure of Juno + CD9 before simulation and after simulation, where we can see that the Juno + CD9 protein complex went through lot of structural changes during the 10 ns molecular dynamics simulation period and gained a coil structure which is probably taking part in protein-protein interaction. Molecular dynamics of proteins reflects the biological process of protein folding unfolding protein-protein interaction, so we can assume that Juno + CD9 exerts the same characteristics in biological system.

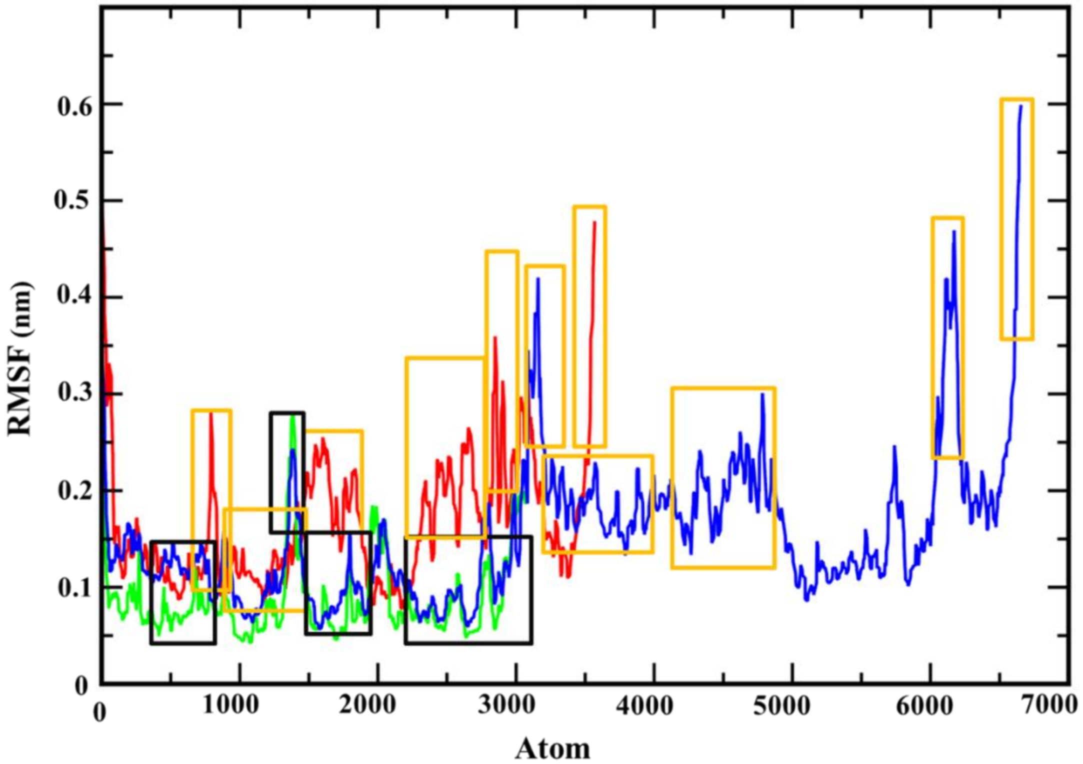

Supplementary Figure S15: RMSF analysis of Juno (green), CD9 (red) and Juno+CD9 (blue). Fluctuation rate of Juno is ~ 2.0 Å, where CD9 possess ~ 2.9 Å and Juno + CD9 ~ 2.5 Å (excluding the c-terminal ~ 6.0 Å). The marked box reflects similar fluctuation patterns where black box representing the Juno and Juno + CD9 RMSF and yellow box CD9 vs Juno + CD9. The RMSF is also indicating that the Juno + CD9 complex reflects biologically favorable interaction processes.

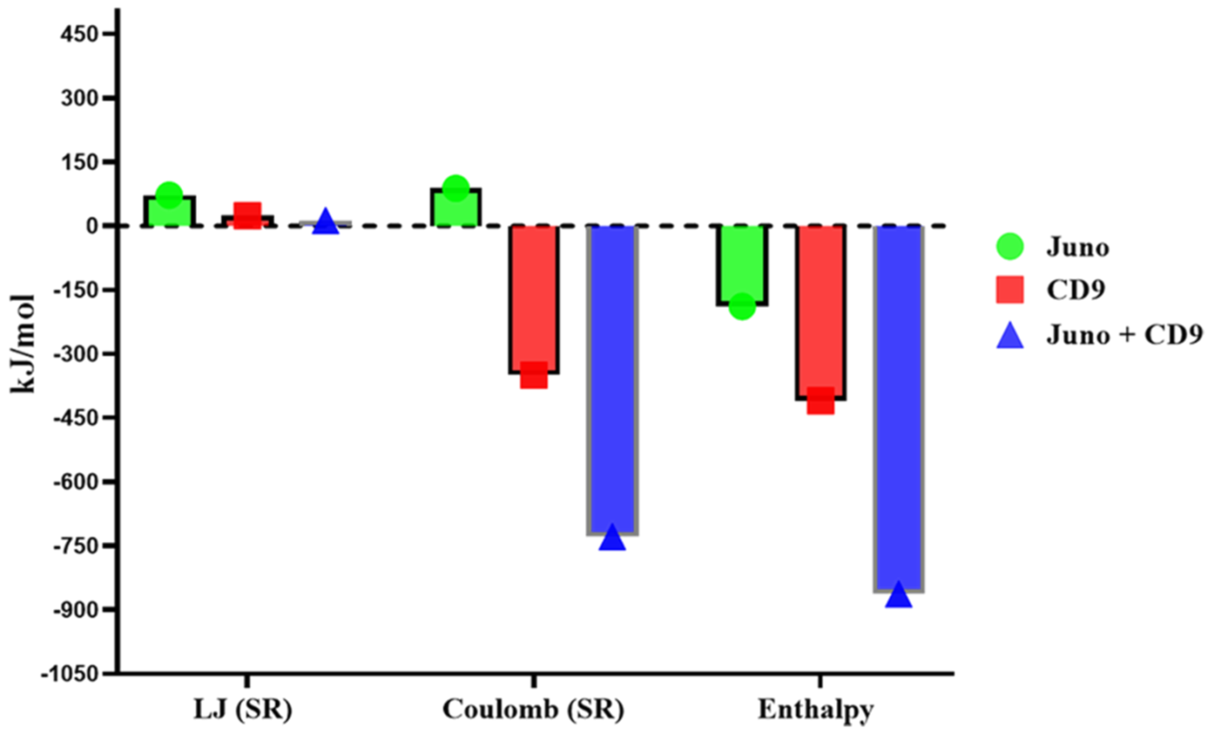

Supplementary Figure S16: Energy profiling for Juno, CD9 and Juno + CD9. The coulombic energy for Juno was low (88.3046 kg/mol) compared to CD9 (-349.117 kg/mol), with the Juno-CD9 complex showing even higher energy (-727.862 kg/mol). CD9 contributes the highest energy towards complex formation, resulting in a stable structure due to a stronger electrostatic force during Juno-CD9 interaction. Short-range Lennard-Jones interaction did not show significant results. The enthalpy values for Juno, CD9, and the Juno-CD9 complex are -188.69 kJ/mol, -410.06 kJ/mol, and -862.06 kJ/mol, respectively. Enthalpy is a crucial factor in protein-protein interactions and is indicative of the overall energy change during the interaction. A favorable enthalpy change signifies an energetically favorable interaction, which contributes to the stability of the complex. The significantly more negative enthalpy for the Juno-CD9 complex suggests a stronger interaction between Juno and CD9 in comparison to their individual states. This information aids in comprehending the underlying mechanisms of protein-protein interactions and may have implications in drug design for targeting such interactions.

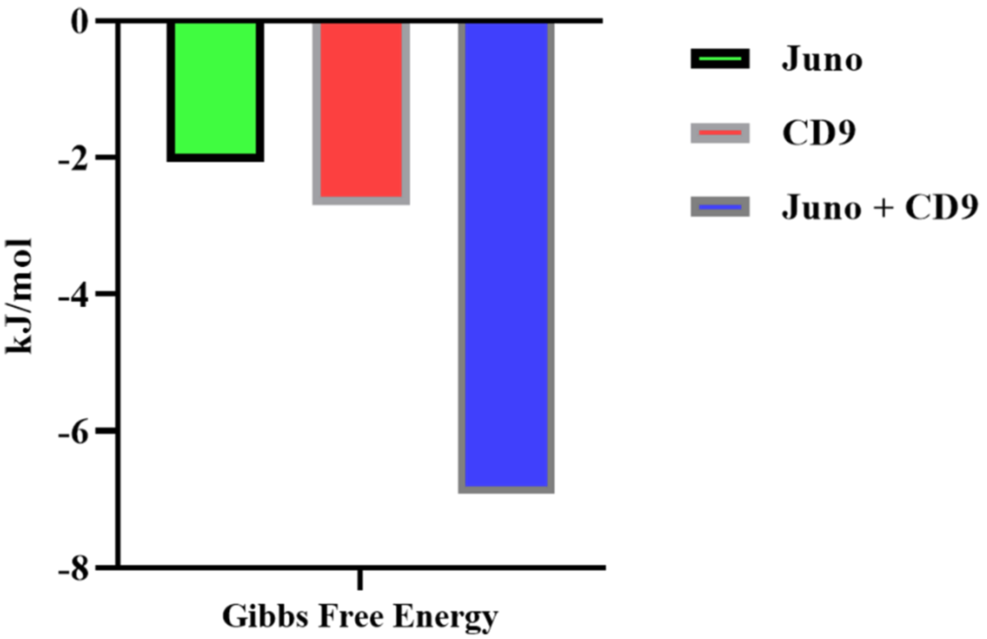

Supplementary Figure S17: Gibbs free energy in the system for dynamic changes and interaction. The Gibbs free energy values for Juno, CD9, and the Juno-CD9 complex are -2.06 kJ/mol, -2.69 kJ/mol, and -6.92 kJ/mol, respectively. These values suggest that the Juno-CD9 complex formation is energetically favorable and thermodynamically stable, indicating a strong interaction between Juno and CD9. These findings provide insights into the thermodynamic profile of protein-protein interactions and may have implications for the development of drugs targeting such interactions.

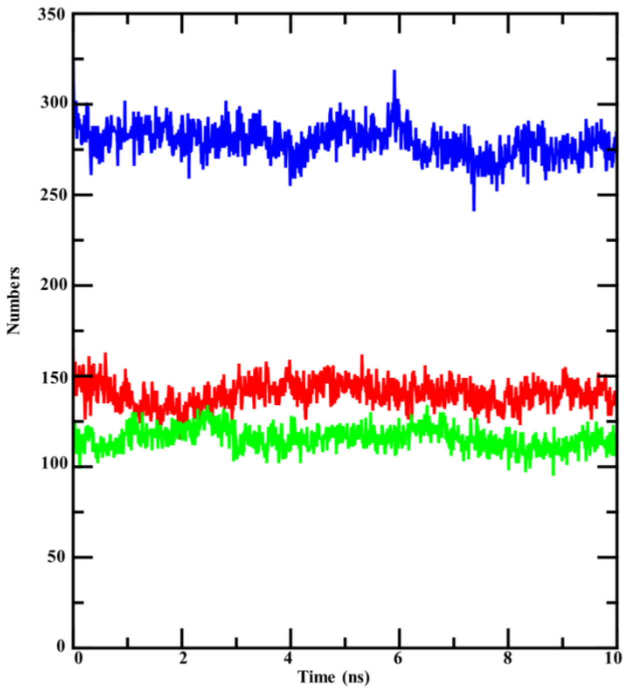

Supplementary Figure S18: Protein-protein hydrogen bonds, where Juno possesses 113 bonds (green), CD9 possesses 126 bonds (red) and Juno + CD9 possesses 278 (blue) bonds end of the 10 ns simulation.

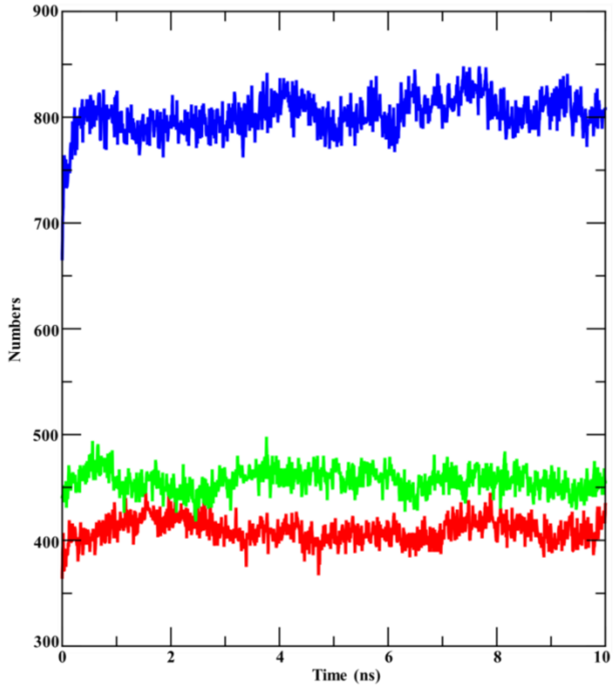

Supplementary Figure S19: Protein-solvent hydrogen bonds, where Juno possesses 455 bonds (green), CD9 possesses 434 bonds (red) and Juno + CD9 possesses 808 (blue) bonds at the end of the 10 ns simulation.

Supplementary Video S1: Visualization of Juno and CD9 localization in oolemma captured by 3D STED.

Supplementary Video S2: Visualization of Juno and CD9 localization in oolemma captured by 3D STED.

Supplementary Video S3: Visualization of mutual position of Juno and CD9 in individual plane of oolemma captured by 3D STED.

Supplementary Video S4: Dynamics of Juno, it can be assumed that in the biological system and solution, Juno movement and motion are similar.

Supplementary Video S5: Dynamics of CD9, it can be assumed that in the biological system and solution CD9 movement and motion are similar.

Supplementary Video S6: Dynamics of Juno + CD9, it can be assumed that in the biological system and solution Juno + CD9 movement and motion are similar.

Supplementary Video S7: Dynamics of Alphafold predicted CD9 from the motion video it can be assumed that the dynamic pattern is similar with our MODELLER predicted CD9.

Reference:

Ohto, U., Ishida, H., Krayukhina, E., Uchiyama, S., Inoue, N., and Shimizu, T. (2016). Structure of IZUMO1–JUNO reveals sperm–oocyte recognition during mammalian fertilization. *Nature* 534**,** 566-569.
